# Flyways and migratory behaviour of the Vega gull (*Larus vegae*), a little-known Arctic endemic

**DOI:** 10.1371/journal.pone.0281827

**Published:** 2023-02-16

**Authors:** Olivier Gilg, Rob S. A. van Bemmelen, Hansoo Lee, Jin-Young Park, Hwa-Jung Kim, Dong-Won Kim, Won Y. Lee, Kristaps Sokolovskis, Diana V. Solovyeva

**Affiliations:** 1 UMR 6249 Chrono-Environnement, CNRS, Université de Bourgogne Franche-Comté, Besançon, France; 2 Groupe de Recherche en Ecologie Arctique (GREA), Francheville, France; 3 Bureau Waardenburg, Culemborg, The Netherlands; 4 Korea Institute of Environmental Ecology (KOECO), Daejeon, Republic of Korea; 5 National Migratory Bird Research Center, National Institute of Biological Resources, Ongjin-gun, Republic of Korea; 6 Division of Life Sciences, Korea Polar Research Institute, Incheon, Republic of Korea; 7 Department of Biology, Lund University, Lund, Sweden; 8 Laboratory of Ornithology, Institute of Biological Problems of the North, Magadan, Russia; Southeastern Louisiana University, UNITED STATES

## Abstract

Large gulls are generalist predators that play an important role in Arctic food webs. Describing the migratory patterns and phenology of these predators is essential to understanding how Arctic ecosystems function. However, from all six large Arctic gull taxa, including three long-distance migrants, to date seasonal movements have been studied only in three and with small sample sizes. To document the flyways and migratory behaviour of the Vega gull, a widespread but little-studied Siberian migrant, we monitored 28 individuals with GPS loggers over a mean period of 383 days. Birds used similar routes in spring and autumn, preferring coastal to inland or offshore routes, and travelled 4000–5500 km between their breeding (Siberia) and wintering grounds (mainly the Republic of Korea and Japan). Spring migration mainly occurred in May, and was twice as fast and more synchronized among individuals than autumn migration. Migration bouts mainly occurred during the day and twilight, but rates of travel were always higher during the few night flights. Flight altitudes were nearly always higher during migration bouts than during other bouts, and lower during twilight than during night or day. Altitudes above 2000m were recorded during migrations, when birds made non-stop inland flights over mountain ranges and vast stretches of the boreal forest. Individuals showed high inter-annual consistency in their movements in winter and summer, indicating strong site fidelity to their breeding and wintering sites. Within-individual variation was similar in spring and autumn, but between individual variation was higher in autumn than in spring. Compared to previous studies, our results suggest that the timing of spring migration in large Arctic gulls is likely constrained by snowmelt at breeding grounds, while the duration of migration windows could be related to the proportion of inland versus coastal habitats found along their flyways (‘fly-and-forage’ strategy). Ongoing environmental changes are hence likely in short term to alter the timing of their migration, and in long term possibly affect the duration if e.g. the resource availability along the route changes in the future.

## Introduction

Most Arctic breeding birds engage in seasonal migrations to maximize their fitness in summer (e.g., by exploiting seasonally abundant resources and reducing nest predation) and to escape the poor feeding and harsh weather conditions prevailing in winter [1–3]. Although migratory flyways and behaviour may provide fundamental information for better understanding of the Arctic ecosystems, they remain poorly known in many taxa.

Whether they are coastal or inland breeders, Arctic gulls are important generalist predators in this biome, especially given the low number of vertebrate predator species found in Arctic coastal and terrestrial ecosystems [4]. They can feed on carrion and on a large variety of prey, from fishes and marine invertebrates to birds and terrestrial arthropods, and even on cyclic lemmings [4–6]. Given their high trophic position, it is important to improve our ecological knowledge of the ecology of Arctic gulls in general, and the phenology of their seasonal movements in particular. For example, documenting the precise timing of their migration is needed to determine the period when they interact with their Arctic prey species whereas knowing their wintering areas and migration flyways is essential to document the challenges and threats they face over the course of their annual cycle, as well as possible carryover effects. The latter can directly impact their annual survival and hence their long-term population dynamics, but also indirectly impact other species within their Arctic communities.

At least six gull taxa of the genus *Larus* can be seen as Arctic endemics, hereafter referred to as “Arctic gulls” [6,7]. With its circumpolar breeding range and larger body size, the Glaucous gull (*L*. *hyperboreus*) is the most widespread species. The other, nearly vicariant taxa, are only breeding in restricted regional ranges: Northern Pacific (Glaucous-winged gull, *L*. *glaucescens*), Canadian Arctic archipelago and Northwest Greenland (Thayer’s gull, *L*. *glaucoides thayeri*), Northwest Atlantic (Iceland gull, *L*. *g*. *glaucoides* from Southeast to West Greenland, *L*. *g*. *kumlieni* in Northeast Canada), Western Russian Arctic between the Kola and Taymyr Peninsulas (Heuglin’s gull, *L*. *fuscus heuglini*) and Eastern Siberia (East of the Taymyr Peninsula, Vega gull, *L*. *argentatus vegae* or *L*. *vegae*). Among these six Arctic gulls, only three taxa (i.e., Thayer’s, Heuglin’s and Vega gulls) typically engage in long-distance migrations, from the Arctic to temperate, sub-tropical or even tropical climatic zones, with distances of 5000 km or more between their breeding and wintering ranges [6,8–10].

Although the migration patterns of four individual Thayer’s gulls have recently been described using Argos satellite tracking [8], similar studies are currently lacking for the two other long-distance migrants, i.e. Heuglin’s and Vega gulls.

Using high-resolution GPS loggers, this study documents and describes the seasonal movements of individual Vega gulls. In particular, we aimed to answer the following questions: (1) what is the timing of their migrations, (2) which flyways they use and is there any migratory connectivity between breeding and wintering sites [11], (3) are they consistent in their migratory movements, (4) at which speeds, altitudes and light regimes do they migrate.

## Material and methods

### Study species

The taxonomic status of Vega gull is still debated and the general knowledge of several aspects of its ecology is lacking. Based on mitochondrial DNA [12], some authors suggested to consider it as a full species (*Larus vegae* Palmen, 1887), as did Clements in its World Checklist [13] between 2002 and 2007. However, in the most recent taxonomies, the Vega gull is either listed as a subspecies of the European (*Larus argentatus vegae*; [7]) or American (*Larus smithsonianus vegae*) Herring gull ([14]; see also [15–17]).

Regardless of this ongoing debate, Vega gull is clearly separable from other gull species, both phenotypically and geographically [12,18,19]. Among the six Arctic gulls, it can only be confused with the Thayer’s gull, which has a similar grey back and black primaries, but this latter species breeds in Arctic North America and winters from British Columbia to Baja California, while the Vega gull breeds in Eastern Siberia (from the Taymyr Peninsula to the Bering Strait), and winters in the coastal regions of Japan, Korea and China [6,12,20]. In this study, the southern limit of the Vega gull’s breeding range was set to 62.5°N based on our own results and on D.V. Solovyeva’s expertise.

### Study sites and trapping

A total of 50 Vega gulls were trapped and tagged over five years at three separate locations. On the breeding grounds, in the Chaun Delta (Chukotka, Russia, 68.833°N, 170.500°E) where the Vega gull is a regular breeder, sometimes in mixed colonies with other gull species [21], trapping occurred in the summers of 2017 (n = 5), 2018 (n = 9) and 2019 (n = 3). In the wintering range, trapping occurred at Samcheok (Gangwon Province, Republic of Korea, 37.390°N, 129.240°E) in February 2015 (n = 12), November 2015 (n = 4) and February 2016 (n = 3), and at Yeongdeok (Gyeongsangbuk Province, Republic of Korea, 36.376°N, 129.401°E) in February 2017 (n = 14). This sequential and multi-site trapping allowed us to simultaneously monitor at least four different individuals (mean: 6.5; maximum: 12) during each month of this five-year study, and at least 15 different individuals per month when years were merged (S1 Table).

In the Chaun Delta, we used hand-made clap nets (diameter: 90cm) on nests or small roosting places (where gulls were attracted by fish bait), and snares (15cm diameter loops made of 1mm fishing line and attached to a metal rope) on the breeding colonies with high nest densities. On the Korean wintering grounds, we used air cannon nets (12x12m net powered by three air tanks; KoEco Inc.) on fish-baited sand beaches.

Once trapped, birds were aged according to their plumage [19] and weighed to the nearest 5 g (except at the Yeongdeok site) before being tagged with GPS-Mobile Phone loggers (see below). Birds from Chaun Delta and Samcheok (in February 2015) were also genetically sexed [22].

### GPS tracking and data filtering

In February 2015, birds were tagged with battery-powered loggers (model WT-200; KoEco Inc., Daejeon, Republic of Korea; http://www.wi-tracker.com/) weighing 57 g (i.e., on average 4.87% of birds’ body mass; range: 3.56–5.48%). For all other birds tagged between November 2015 and June 2019 we used three different types of solar-powered GPS logger (model WT-300, WT-300 Buzzard and WT-300 Mallard; KoEco Inc.) weighing between 27 and 42 g (i.e. on average 2.87% of birds’ body mass; range: 2.10–3.94%; [23]). Loggers were attached on the birds with a backpack harness weighing ca. 3 g and made of Teflon ribbon and silver rings [24].

Duty cycle of the loggers was set to record one fix every 12 h for the large WT-200 model deployed in February 2015, and one fix every 2 or 4 h for all others (Table 1). GPS positions were stored in the loggers until birds came within reach of a mobile phone network, where they were automatically uploaded (daily) on a web server (“Wild Tracking System”, KoEco Inc.; [25]), allowing us to access, visualize, and download the data.

**Table 1:** Summary data for the 28 Vega gulls included in this study.

Trapping location and individual ID	Age (sex from DNA when stated)	GPS logger^a^	GPS Start Date (UTC+9)	GPS End Date (UTC+9)	No. days	No. of filtered GPS fixes	Duty cycle (fixes/day)	Total track (km)	Mean distance travelled per day (km)	Longest great circle dist. (km)	Wintering region (1 Dec.-31 March)	Summering region (1 June-31 August) (BR = within breeding range; OBR = outside b. range)
**Samcheok (Gangwon Province), South Korea**												
ke1511	2nd year (male)	WT-200	25/02/2015	26/10/2015	243	487	2	6473	26.6	2252	E Coast, South Korea	N Sakhaline Is., Russia (OBR)
ke1512	Adult (female)	WT-200	25/02/2015	19/11/2015	267	534	2	12447	46.6	4123	E Coast, South Korea & W Japan	Chromskaya Bay, N Yakutia, Russia (BR)
ke1513	Adult (male)	WT-200	25/02/2015	22/07/2015	147	286	2	1860	12.7	609	E Coast, South Korea	Russian-N Korean border (OBR)
ke1514	Adult (male)	WT-200	25/02/2015	29/10/2015	246	493	2	13022	52.9	4226	E Coast, South Korea	Olvegyrgyvaam R, NW Chukotka, Russia (BR)
br1425	Adult (male)	WT-200 / 300^b^	25/02/2015	15/11/2016	629	5041	2 / 12^b^	16349	26.0	3958	E Coast, South Korea (x2)	Alazeya R basin, NE Yakutia, Russia (BR)
br1428	Adult (male)	WT-200	25/02/2015	02/10/2015	219	439	2	16480	75.3	4232	E Coast, South Korea	Malaya Balakhnya R, SE Taimyr, Russia (BR)
br1429	Adult (female)	WT-200	25/02/2015	11/05/2016	441	885	2	18846	42.7	4019	E Coast, South Korea & W Coast, North Korea	Putorana Plateau, Russia (BR)
br1430	Adult (female)	WT-200	25/02/2015	22/11/2015	270	540	2	12596	46.7	3666	E Coast, South Korea	Kolyma R, E Yakuta, Russia (BR)
vt15079	Adult	WT-300	09/11/2015	05/05/2017	543	6457	12	26282	48.4	4341	E Coast, South Korea (x2)	Olenek Delta, NW Yakutia, Russia (BR)
vt15081	Adult	WT-300	09/11/2015	11/05/2017	549	4734	12	27155	49.5	4346	E & S Coasts, South Korea (x2)	Gusinnaya River, N Yakutia, Russia (BR)
vt15088	Adult	WT-300	09/11/2015	15/04/2016	158	2103	12	10908	69.0	3402	E Coast, China & E Coast, South Korea	Sakhaline Is., Russia (OBR)^g^
br1530	2nd year	WT-300 Mallard	16/02/2016	30/04/2017	439	2661	6	20459	46.6	3367	E & SE Coast, South Korea	Sea of Okhotsk coast, Russia (OBR)
br1541	Adult	WT-300 Mallard	18/02/2016	14/05/2018	816	3776	6	42615	52.2	4286	E Coast, South Korea & W Japan	Malaya Kuropatochiya R, NE Yakuta, Russia (BRx2)
**Yeongdeok (Gyeongsangbuk Province), South Korea**												
vt16247	Adult	WT-300	13/02/2017	10/10/2018	604	6866	12	29933	49.6	4009	E Coast, South Korea	Yana Delta, N Yakutia, Russia (Bx2)
vt16249	Adult	WT-300	13/02/2017	30/05/2017	106	1237	12	8470	79.9	3428	E Coast, South Korea	Yakutia, Russia (BR)^g^
vt16250	Adult	WT-300	13/02/2017	19/02/2019	736	8740	12	38211	51.9	4106	E Coast, South Korea (x2)	Shadrin R, NE Yakutia, Russia (BRx2)
vt16251	Adult	WT-300	13/02/2017	27/05/2017	103	1197	12	7491	72.7	3254	E Coast, South Korea	Bering Sea cost, E Chukotka, Russia (BR)^g^
vt16252	Adult	WT-300	13/02/2017	27/04/2019	803	9551	12	46484	57.9	3974	E Coast, South Korea (x2)	Alazeya R, E Yakutia, Russia (Bx2)
vt16254	Adult	WT-300	13/02/2017	06/06/2017	113	1263	12	5631	49.8	2187	E Coast, South Korea	N Sakhaline Is., Russia (OBR)
vt16260	1st year	WT-300	13/02/2017	25/06/2017	132	1532	12	7357	55.7	3263	E Coast, South Korea	Magadan, Russia (OBR)
vt16261	1st year	WT-300	13/02/2017	19/09/2017	218	2615	12	6965	31.9	2230	E Coast, South Korea	N Sakhaline Is., Russia (OBR)
**Chaun Delta (Chukotka Region), Russia**												
bpn1721	Adult male	WT-300	24/07/2017	31/12/2019	890	9912	12	51010	57.3	4942	S & SE Coast, South Korea	Chaun Delta, Russia (BR)
bpn1723	Adult male	WT-300	22/07/2017	10/05/2018	292	3487	12	10901	37.3	4108	Tone River estuary, E Japan	Chaun Delta, Russia (BR)
rcees1809	Adult female	WT-300	28/06/2018	31/12/2019	551	5988	12	30490	55.3	4738	S Coast, South Korea	Chaun Delta, Russia (BR)
rcees1811	Adult female	WT-300	28/06/2018	29/05/2019	335	3676	12	20275	60.5	4840	S Coast, South Korea	Chaun Delta, Russia (BR)
rcees1812	Adult male	WT-300	28/06/2018	31/12/2019	551	6581	12	19505	35.4	4327	Honshu (NE & W coasts) & Hokkaido^d^, Japan	Sakhaline Is. (OBR) and Chaun Delta, Russia (BR)
bpn1910	Adult female	WT-300 Buzzard	23/06/2019	14/11/2019	144	1783	12	8440	58.6	3512	S Hokkaido, N Japan^e^	Chaun Delta, Russia (BR)
bpn1911	Adult female	WT-300 Buzzard	23/06/2019	31/12/2019	191	1153	12	7185	37.6	4114	Kamisu, E Japan^f^	Chaun Delta, Russia (BR)
Summary data:	28 individuals		25/02/2015	31/12/2019	10736	94017		523840	48.8	4942		

^a^ KoEco WT-200: 57g battery powered; KoEco WT-300: 42g solar; KoEco WT-300 Buzzard: 34g solar; KoEco WT-300 Mallard: 27g solar.

^b^ bird br1425 was recaptured at the same wintering location on 9 November 2015 when the GPS logger was replaced (new duty cycle = 12 positions per day).

^c^ additional locations collected after this date but not included in this study.

^d^ this bird was still on Hokkaido island on 31 Dec. 2019, but wintered on Honshu from January to April 2020.

^e^ location of last positions collected in 2019; in March 2020 this bird was still in S Hokkaido.

^f^ mid-January 2020 this bird was located at Nagoya, i.e., 400 km to the SW of Kamisu.

^g^ location of last positions collected in April-May, i.e. before reaching breeding sites.

Between February 2015 and December 2019, we collected ca. 115,000 GPS positions from the 50 tagged birds. Birds with less than 100 days of monitoring (n = 22) were removed from the analyses because these tracks were too short to document seasonal movements between breeding and wintering areas. The fates of birds were inferred following a classification tree adapted from Sergio et al [26] (S4 Table, S7 Fig). Among the 22 birds that were not used in our analyses, one likely died or lost its transmitter (stationary positions at the end of monitoring), we suspect technical failure of two loggers (i.e., number of positions collected ≤80% of expected and/or voltage ≤3.9V), while the 19 remaining birds had unknown fates (no evidence of technical failure or stationary behaviour). Among the 28 birds whose tracks were used in the following analyses (i.e., with more than 100 days of monitoring), four continued to record locations after the end of the study (31^st^ December 2019), we suspect technical failure for six birds, and the remaining 18 birds had unknown fates but were moving until the very last positions were received.

Furthermore, 1045 positions were removed because they were failed attempts (i.e., latitude and longitude set to 0/0), and 474 because they were duplicate positions. A total of 94,017 positions (form 28 birds) remained after filtering and were used in this study.

### Data analyses

Among the 28 gulls included in the filtered dataset, 24 were adults (seven females, seven males, 10 of unknown sex) and four were immatures (Table 1). Seven birds (including the four immatures) remained in coastal habitats south of 60°N (i.e., outside the species’ breeding range). These latter seven birds, as well as the 2^nd^ year of data for one adult (#rcees1812) tagged at the breeding site but which remained south of 60°N in the following year, were excluded from most analyses hence performed on only 21 adult individuals (subsample A; S4 Table).

Following Cohen et al. ([27]) decision tree, we used Mantel tests implemented in the R package “MigConnectivity” [28] to assess migratory connectivity for the same 21 individuals (subsample A). Confidence intervals (95%) were estimated based on 1000 bootstraps.

To reduce the bias inherent to the sinuosity of the tracks (see e.g. [29–31]) and to the use of different duty cycles (or to failed location attempts), “rates of travel” (i.e., distance between fixes) were always estimated by using only fixes collected over standardized time steps (i.e., two hours or one day), except in S1 Fig which aimed to consider the different duty cycles used. To estimate the individual repeatability of routes and to map individual tracks, we normalized the tracks to daily intervals using the function “redistltraj” in the adehabitat package [32] in R [33]. Conversely, when the aim was only to document temporal changes in distribution, or extreme values (e.g., earliest/latest date or arrival/departure), we used all available fixes. To calculate great-circle distances between polar coordinates (and average positions), we used standard great circle equations [34].

Following similar studies, the migration phenology was described using the absolute displacement method combined with a spatial threshold [35–39]. We defined an “active migration day” if the bird covered a daily distance of more than 60 km and was located between the northern limit of its wintering range (i.e., 40.9°N) and the southern limit of breeding sites used by Vega gulls in our study (i.e., 64.9°N). The threshold distance was inferred from recorded speeds (S1 Fig) and similar studies (e.g., [35]), while the latitudinal boundaries were defined after exploring the spatiotemporal distribution of all birds over the five years of the study (S2 Fig). This definition does not mean however that birds cover 100% of their migratory route during active migration days, they can also cover significant distances by simply moving between successive stopover sites at smaller daily rates of travel. For each spring and autumn migrations, we estimated a “population migratory window” defined as the period (in days) that elapsed between the first and last recorded day of active migration (all birds combined). The same definition was followed to document “individual migratory windows”.

To document the migratory behaviour of each gull and test for possible differences between spring and autumn migrations, we considered only the 13 individuals (subsample C; S4 Table) that were monitored during entire migratory windows in spring and autumn of the same year (i.e., which were located at least once before the onset of spring migration and once after the end of autumn migration). This reduced the sample size but allowed using Wilcoxon signed rank tests to compare medians between paired spring and autumn data. For individuals that produced two years of data (n = 3), we only considered the first year in the analyses.

For the following analyses of diel activity patterns, rates of speed and altitudes, we used the data obtained from the 15 individuals whose loggers had a 2h duty cycle, excluding all bouts shorter than 1.9 and longer than 2.1h (subsample B; S4 Table). We refer to “migration bouts” where birds had moved more than 5 km during a 2h period.

We inferred diel activity patterns according to solar angles (corrected for atmospheric refraction effects: www.esrl.noaa.gov/gmd/grad/solcalc), with “night” when the solar elevation angle was below 6 degrees (i.e., civil twilight), “day” when above 6 degrees, and “twilight” for bouts that started at “night” and ended at “day”, or vice versa.

We used altitudes produced by GPS devices. Accuracy of bird altitudes measured with GPS loggers has been reported to range within a few meters (e.g., 2.77m, 95% CI: 0.38–7.61 m [40]) and is hence appropriate to document large differences in flight altitudes. The mean altitude of each “migration bout” (i.e., a 2 h—leg) was estimated by averaging the start and end altitudes of this bout (after setting negative altitudes to zero).

We used Mann-Whitney U-tests to investigate possible differences in altitudes and rates of travel between months, diel patterns (i.e., day, twilight or night as defined below) and activity (i.e., migration bouts versus other bouts).

For mapping and analyses of individual repeatability, we also used the regularized tracks (see above) from the 28 individuals. Subsequently, positions were projected using a Lambert azimuthal equal-area projection and utilization kernels were estimated across all individuals but using only the first year of data for each individual in order to give each bird the same weight. Separate kernels, using a smoothing factor of 100 km, were estimated for four periods: winter (18 December 12 April), spring (13 April– 6 June), summer (7 June– 31 August) and autumn (1 September– 17 December).

As a measure of individual spatial consistency of movements between years, we used Dynamic Time Warping (DTW) to compare tracks from different years, either both from the same individual or from different individuals. DTW was calculated using the “SimilarityMeasures” package in R [41] with a statistical significance threshold of 0.05. DTW algorithms compare two temporal sequences (e.g., two bird tracks) by searching the path (called *wrapping path*) between two tracks with the smallest cumulative distance between them. Larger DTW values indicate greater dissimilarity between tracks. DTW can be used to compare tracks having different speeds and lengths, and is hence very suitable for animal tracking data, but it is sensitive to both geometry and distance [42]. This was repeated for each of the four seasons mentioned above. The statistical significance between groups of DTW values was assessed by randomization procedures with 10,000 permutations. In each permutation, the group labels were randomly redistributed, and the difference in their means calculated. Subsequently, we assessed how often the observed difference between the two groups was smaller than or larger than the randomized difference in means.

Too few individuals were tracked to quantify the individual repeatability statistics in timing. We do however present the absolute consistency in timing, i.e., the difference in day-of-year between the first and second year of tracking at the start and the end of spring and autumn migration.

### Ethics statement

Republic of Korea: the study was approved by the Committee of Animal Care and Management of the local government (Permit Numbers: 2015–965, 2016–1634, 2017–205) under the Wildlife Protection and Management Act (Act No. 15835).

Russian Federation: the study is exempt from approval from any Russian authority because catching and handling of animals not listed under the Red Data Book of the Russian Federation or the regional Red Data Books is allowed without permits (Article 44 of Federal Law 52-ФЗ from 24.04.1995 with additions from 2020).

## Results

Following the filtering procedure presented above, a total of 94,017 positions documenting more than 500,000 km of individual tracks for 28 Vega gulls were used for our analyses (Table 1). On average, each gull hence produced 3358 positions (range: 286–9912) over a mean monitoring period of 383 days (range 106–890) and travelled a total distance of 18,709 km (range: 6531–51,010; Table 1).

For all but one bird, the longest distance between summer and winter locations was more than 2000 km (mean 3709 km; S.D. 940 km; range: 609 to 4942 km). Seven individuals (out of 21 tagged on their wintering grounds), including the four immatures, spent their summer outside the breeding range (Table 1).

Although the GPS devices we used were relatively heavy (2.10–5.48% of birds mass; mean: 3.54%), we found no relation between the relative weight of the logger (i.e., mass of tag+harness over body mass) and the mean distance travelled by day (linear regression: *r*^2^ = 0.056) or the number of days birds were monitored (*r*^2^ = 0.059).

### Flyways

The Vega gulls in this study showed weak migratory connectivity (Rmantel = -0.002, 95% CI = -0.15–0.24).

**Fig 1:**
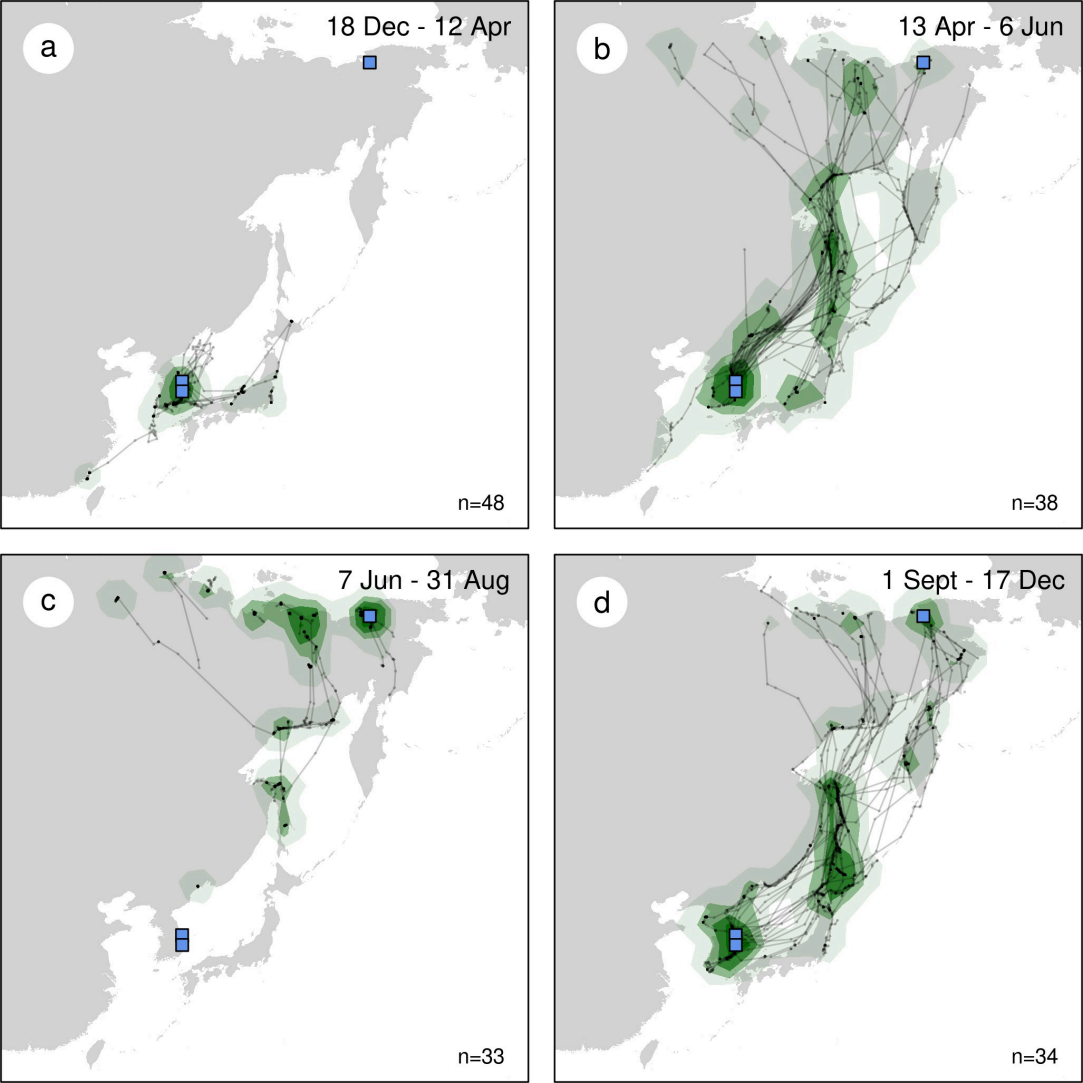
Individual tracks (black lines) and seasonal Kernel Utilisation Distribution (KUD; smoothing factor: 100 km) maps based on the movements of 28 Vega gulls. Blue squares show trapping sites. The four panels present the four seasons of the Vega gull annual cycle (a winter, b spring, c summer, d autumn), with spring and autumn defined as the periods when birds migrate (see S1 Fig (DC = 2) and [35]). The map used as the background was downloaded from http://thematicmapping.org/downloads/world_borders.php and has a Creative Commons Attribution license CC BY-SA 3.0.

Overall, they used relatively parallel flyways for their spring (Figs 1B and S2) and autumn migrations (Figs 1D and S2), with few inland flights except in the northernmost parts of the flyways (i.e., north of the Sea of Okhotsk) where the only other option to reach their breeding grounds would be to engage in a long detour around the Bering Strait. Also, birds remained coastal (i.e., within 10 km of the coastline) for most of the year and avoided flying offshore (i.e., farther than 50 km of the coastline) unless they had no other alternative (e.g., to fly between Japan and South Korea or to shortcut the western part of the Sea of Okhotsk, mainly in autumn). Note that the Kuril Islands, although too small to be seen on the maps, were used by four birds as a dotted land bridge between the Kamchatka Peninsula and Hokkaido (see S3I, S4E and S5DG Figs).

Some differences did however exist between spring and autumn flyways. For example, in spring 80% of the tracks followed the western coast of the East Sea along the North Korean and Russian continental coasts (i.e., along Sakhalin Island), compared to only 20% that went along the Kamchatka Peninsula (Fig 1B). In autumn, a larger proportion (especially the birds tagged in the Chaun Delta) migrated along the Kamchatka Peninsula (33% of all tracks) before reaching Japan or the south-western shores of the Sea of Okhotsk (Fig 1D). Finally, offshore bouts were also less common in spring, except in the western part of the East Sea where most birds preferred to fly offshore to shortcut the coastline of the Korean Peninsula and the Gulf of Peter the Great (near Vladivostok). It is also worth mentioning that (1) Northern Sakhalin Island hosted several of the non-breeding birds in summer (Figs 1C and S6) and that (2) while most birds flew along the coasts of this island during their migrations, they seemed to prefer the western coast during their northbound migration and the eastern coast in autumn (see e.g., S3D and S3E Fig).

The distance from the Korean Peninsula (where 24 out of the 28 birds were wintering) to the southern coast of the Magadan region (ca. 60°N), where most birds passed by and most immatures and non-breeding birds spent their summer (S3 Fig), equals to 3,000–3,500 km (depending on the exact departure point from the Korean Peninsula) for a bird following the above-described flyway on a straight flight. Flying along Japan adds ca. 500 km to this flyway, and up to 750 km for birds using the easternmost route over the Kuril Islands and the Kamchatka Peninsula. Reaching the breeding grounds above the Arctic circle from the southern coast of the Magadan region requires flying another 1,000–2,000 km depending on the final destination. Hence, to migrate to/from the breeding grounds, the bird must cover a distance of 4,000–5,500 km. This is very close to the longest great-circle distance observed between the wintering and breeding grounds of most birds from this study (Table 1), meaning that the flyways used by birds are rather well optimized although they mainly follow coastal lines.

Most birds wintered along the East coast of the Korean Peninsula (Fig 1A), but this greatly reflects the localization of the two Korean winter trapping sites. Indeed, among the seven birds sampled in the Chaun Delta in summer, four remained in Japan in winter (Table 1; S5 Fig). For birds wintering further south along the coast of China (as bird #vt15088; S6 Fig), an additional leg of 500–1,500 km is required to link wintering and breeding grounds.

### Seasonal movements in space and time

**Fig 2:**
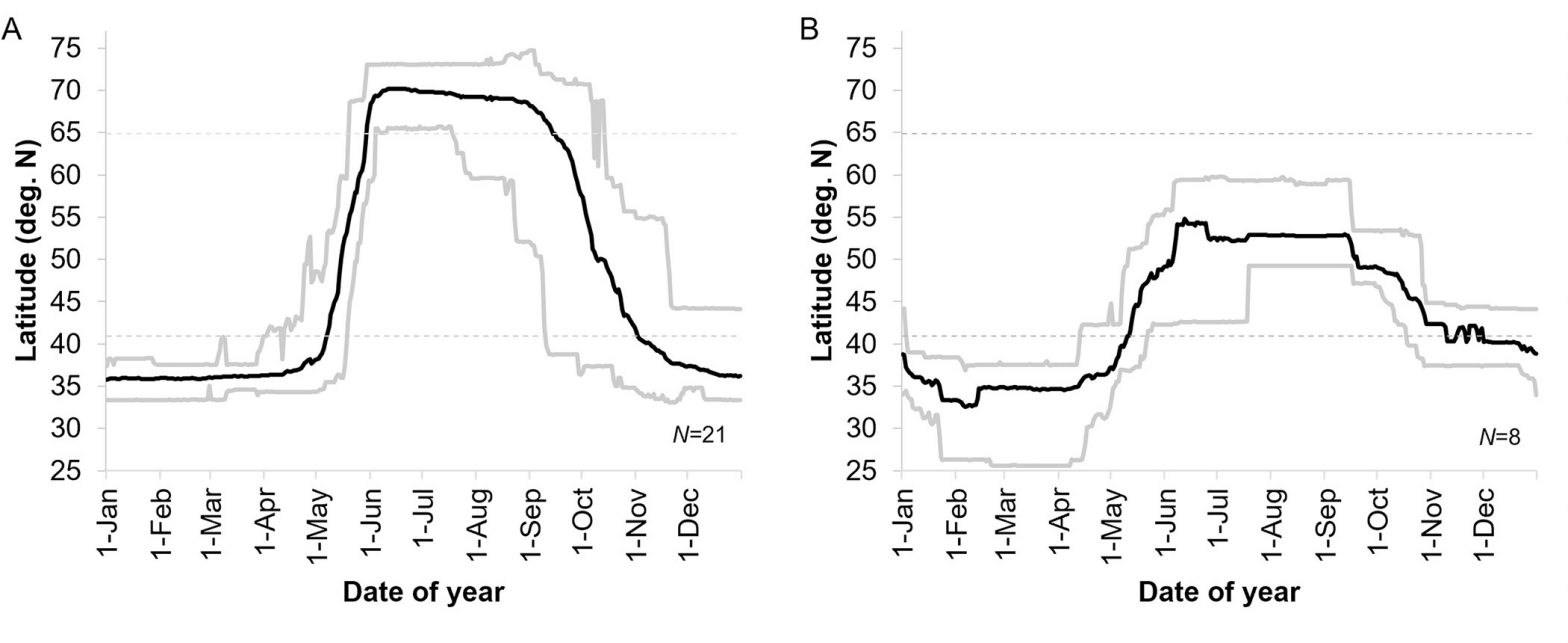
Seasonal changes in the latitudinal distribution of Vega gulls spending summer (A) within (above 64.9°N) or (B) outside (below 60°N) their known breeding range (the fixes of one bird were split between the two panels; see Methods). The black line shows the mean latitude of all birds for each day of the year while the grey lines show the maximum and minimum latitudes recorded during these days for single birds.

**Fig 3:**
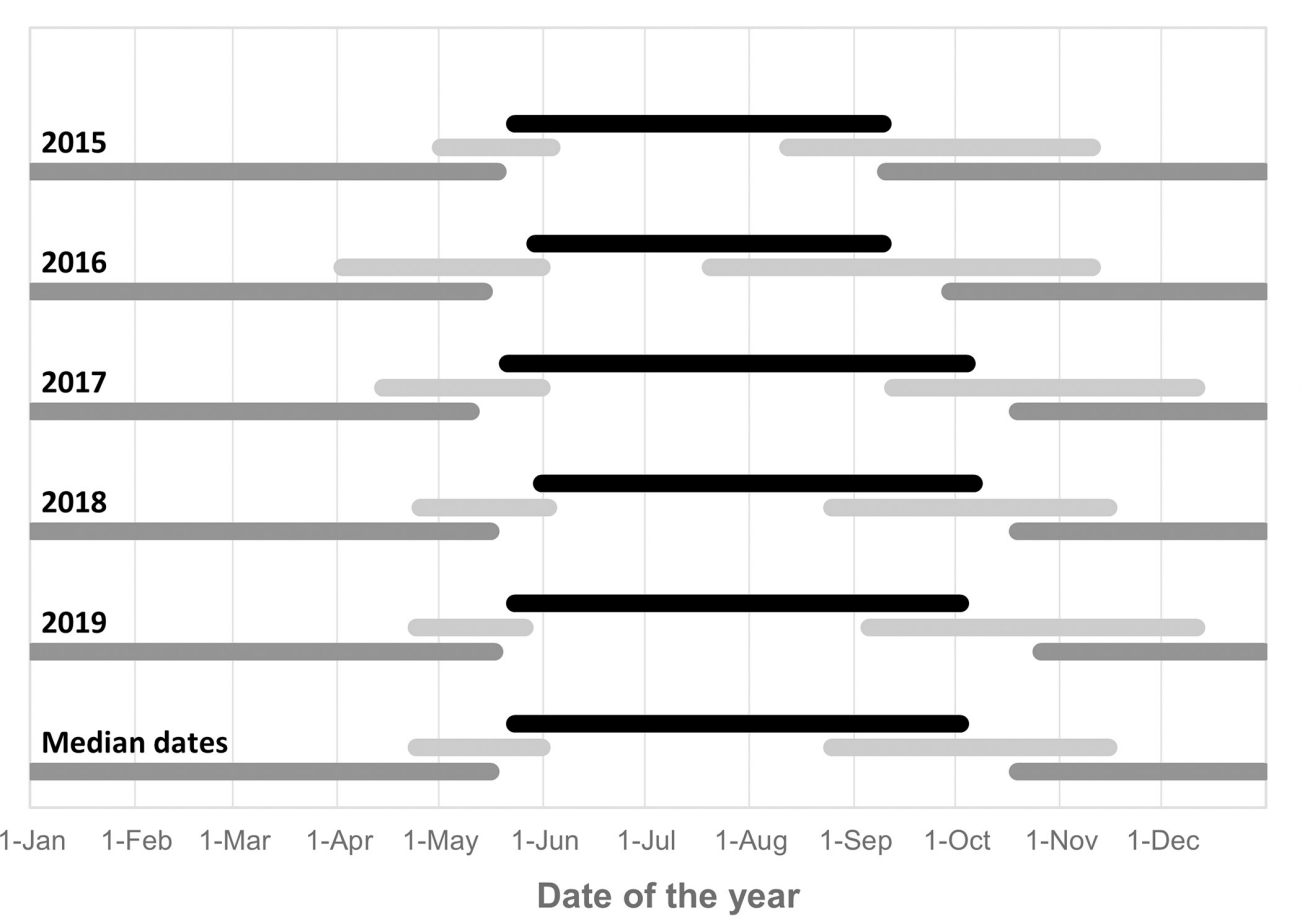
Annual phenology of 21 Vega gulls summering within the breeding range between 2015 and 2019. Lines represent the extend of breeding (black), migratory (light grey), and wintering (dark grey) periods (exact dates are given in S2 Table).

Although one bird (#br1541) departed from the wintering area (south of 40.9°N) on the 2^nd^ of April, in 2016 (but remained below 43° N until the 3^rd^ of May), all others left in late April (median date over 5 years: April 24) and six birds departed as late as mid-May in some years. Spring migration was hence most intense in May and by early June all birds had reached their summering area (the latest date of the spring migration was the 3^rd^ of June, in 2015; median date for all individual estimates: May 31) (Figs 2 and 3; S2 Table).

During summer, there was a strict spatial discrimination between birds that had reached their breeding range, and birds that hadn’t, with no birds staging in between 60 and 64.9°N from the 3^rd^ of June to the 29^th^ of July (Fig 2).

The autumn migration was less synchronized between years and lasted on average twice as long as the spring migration (mean duration: 84 versus 41 days respectively), with the earliest birds leaving their breeding range between 20^th^ of July (in 2016; likely to be failed breeders) and 12^th^ of September (in 2017; median date: August 25). Autumn migration lasted until the 10^th^ of November in 2015 and 2016, and until the 11^th^ of December in 2017 and 2019 (median date: November 15) (Figs 2 and 3; S2 Table).

### Migratory behaviour

**Fig 4:**
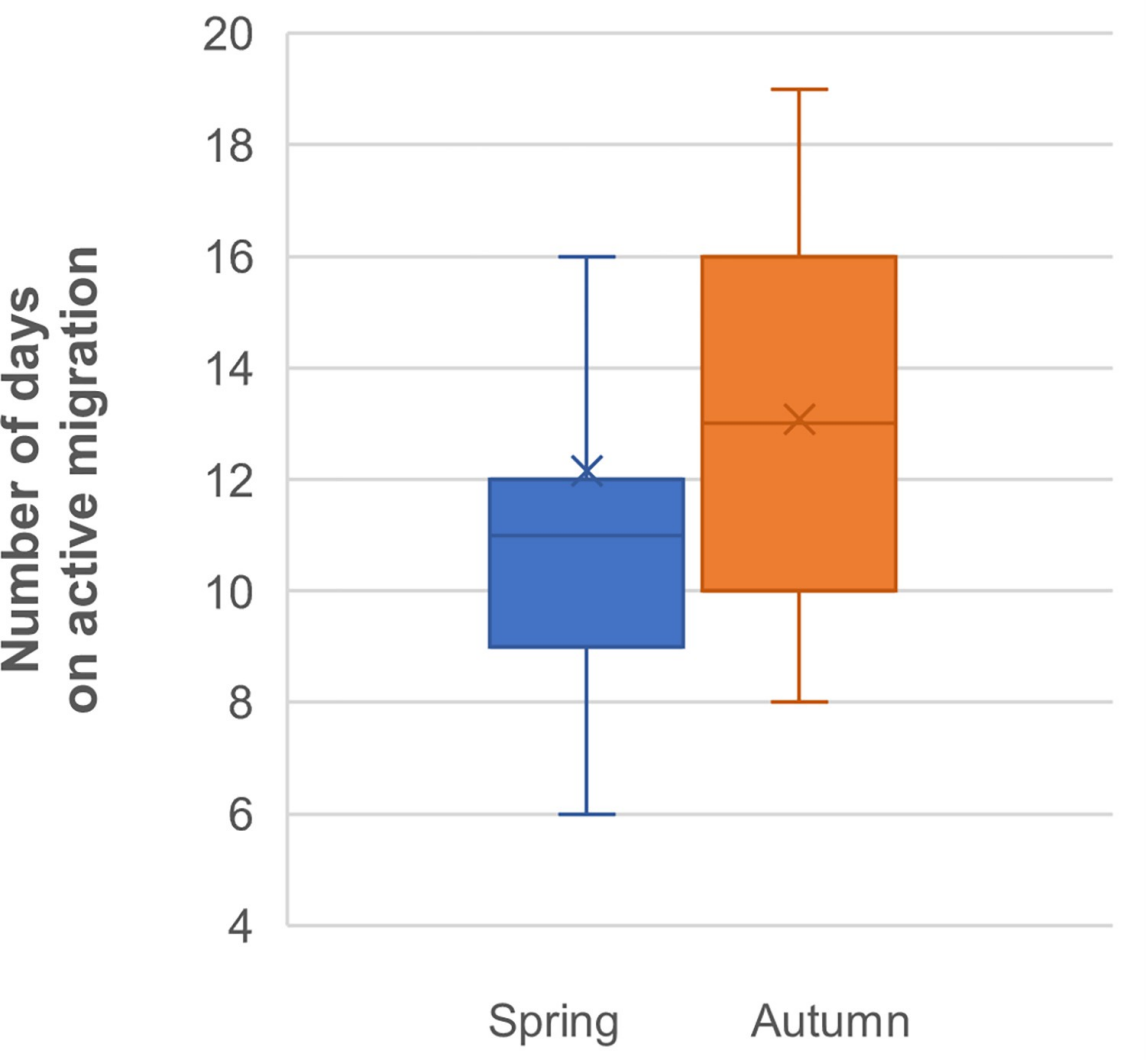
Days of active migration in spring (blue) and autumn (ochre) for 13 Vega gulls. Boxes hold 50% of the values, with the median shown as a horizontal line and the mean as an ‘x’. Whiskers extend to the highest and lowest values excluding outliers.

The median number of days spent on active migration (i.e., more than 60 km travelled per day between 40.9 and 64.9°N) did not significantly differ between both seasons (Z = -0.52, *P* = 0.60; Fig 4), even when corrected for missing days of monitoring (Z = -0.91, *P* = 0.36).

**Fig 5:**
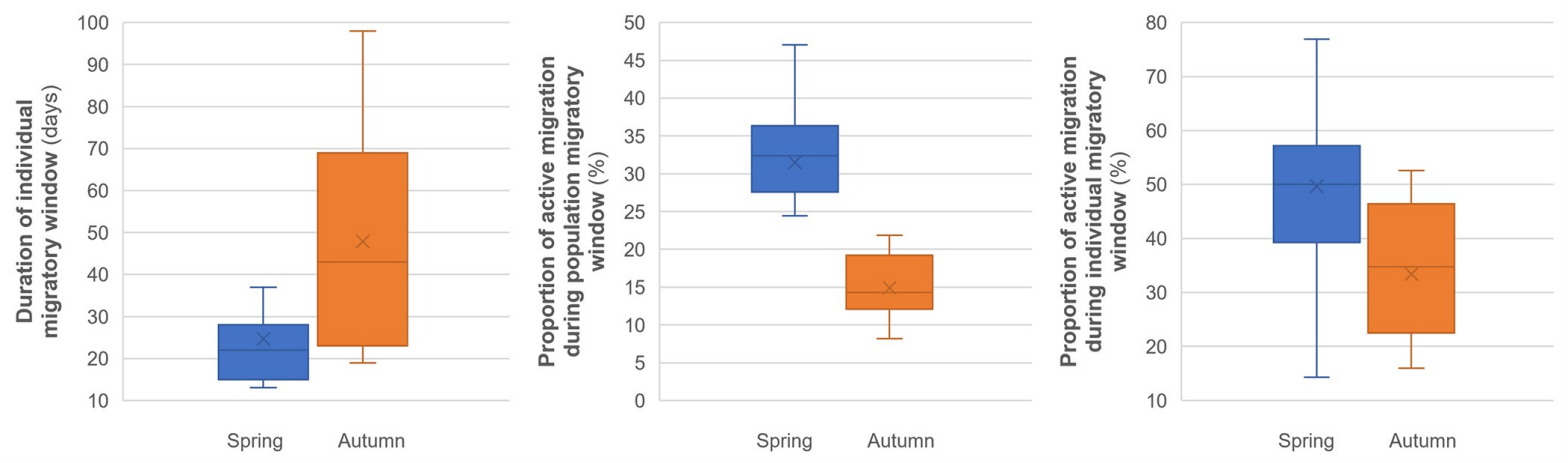
Duration of individual migratory windows of 13 Vega gulls during spring and autumn migrations (A), and proportion of days of active migration during population (B) and individual (C) migratory window. Boxes hold 50% of the values, with the median shown as a horizontal line and the mean as an ‘x’. Whiskers extend to the highest and lowest values excluding outliers.

As already observed at the population level (Fig 2A), the duration of the migratory window was also shorter in spring than in autumn when assessed at the individual level (Wilcoxon signed-rank test: Z = -2.63, *P* = 0.009; Fig 5A). However, during the population migratory windows Vega gulls were on active migration only for 15% of the days in the autumn compared to 32% in the spring (Z = -3.04, *P* = 0.002; Fig 5B), and only 33% in the autumn compared to 50% in spring during their own individual migratory windows (Z = -2.17, *P* = 0.03; Fig 5C).

**Fig 6:**
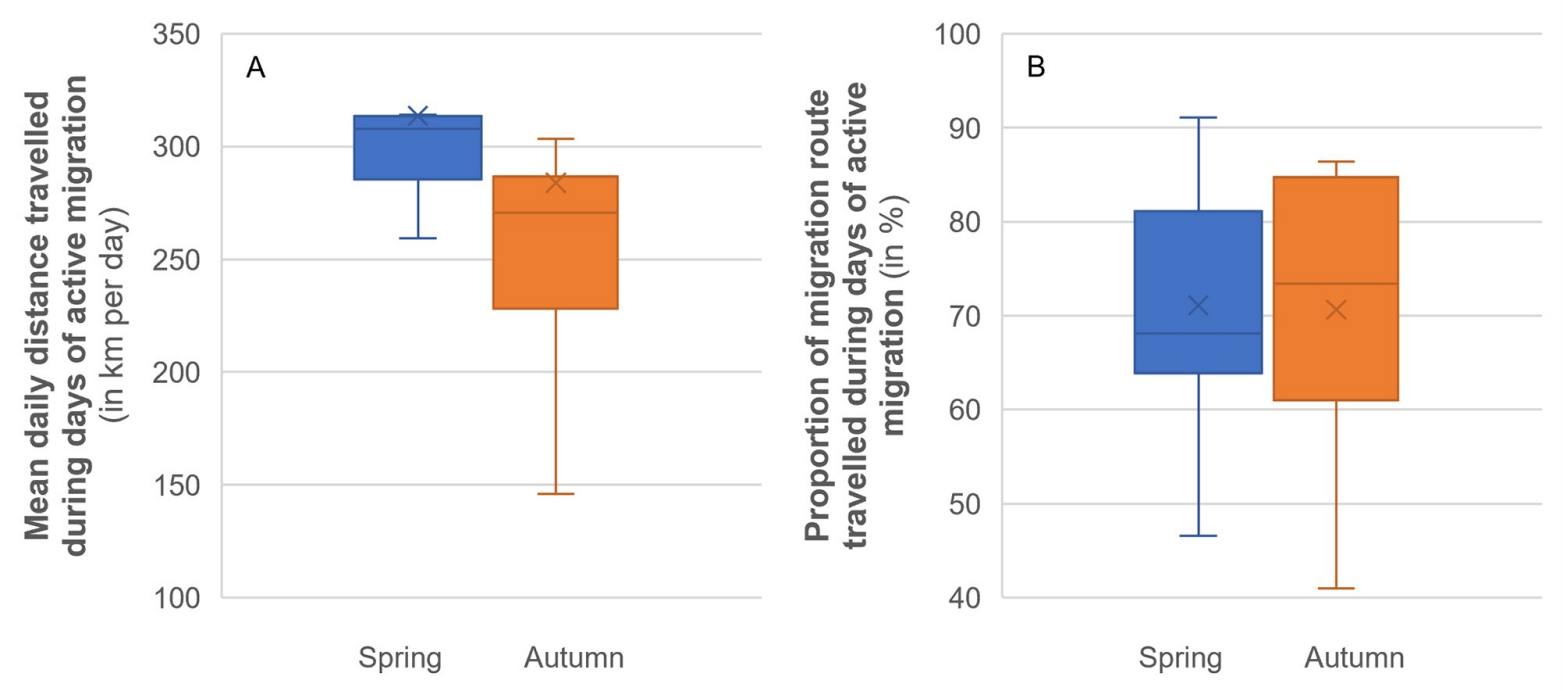
Daily distances (left) and proportion of the migration route travelled during days of active migration (right) by 13 Vega gulls in spring (blue) and autumn (ochre). Boxes hold 50% of the values, with the median shown as a horizontal line and the mean as an ‘x’. Whiskers extend to the highest and lowest values excluding outliers.

Conversely, the mean distances travelled during days of active migration were similar during spring (mean: 314 km) and autumn (mean: 284 km) and did not differ by their median (Z = -0.94, *P* = 0.35; Fig 6A). Although the individual migratory window was twice as short in spring as in autumn (see above), more than two thirds of the distance that birds must travel between winter and summer grounds was covered during the days of active migration (i.e., at high rates of travel), with no difference between seasons (Z = -0.08, *P* = 0.94; Fig 6B). Since the length of the flyway ranges between 4000 and 5500 km (see Results), this means that on average ca. 1000 to 1500 km were covered during the days which are not classified as active migration days (‘fly-and-forage’ strategy).

In conclusion of this section, despite the duration of their spring migration being twice shorter than in autumn, Vega gulls do not travel longer daily distances in spring (Fig 6), they just spend more days on active migration in spring compared to autumn (Fig 5).

### Diel migration pattern, rates of travel and flight altitudes

**Fig 7:**
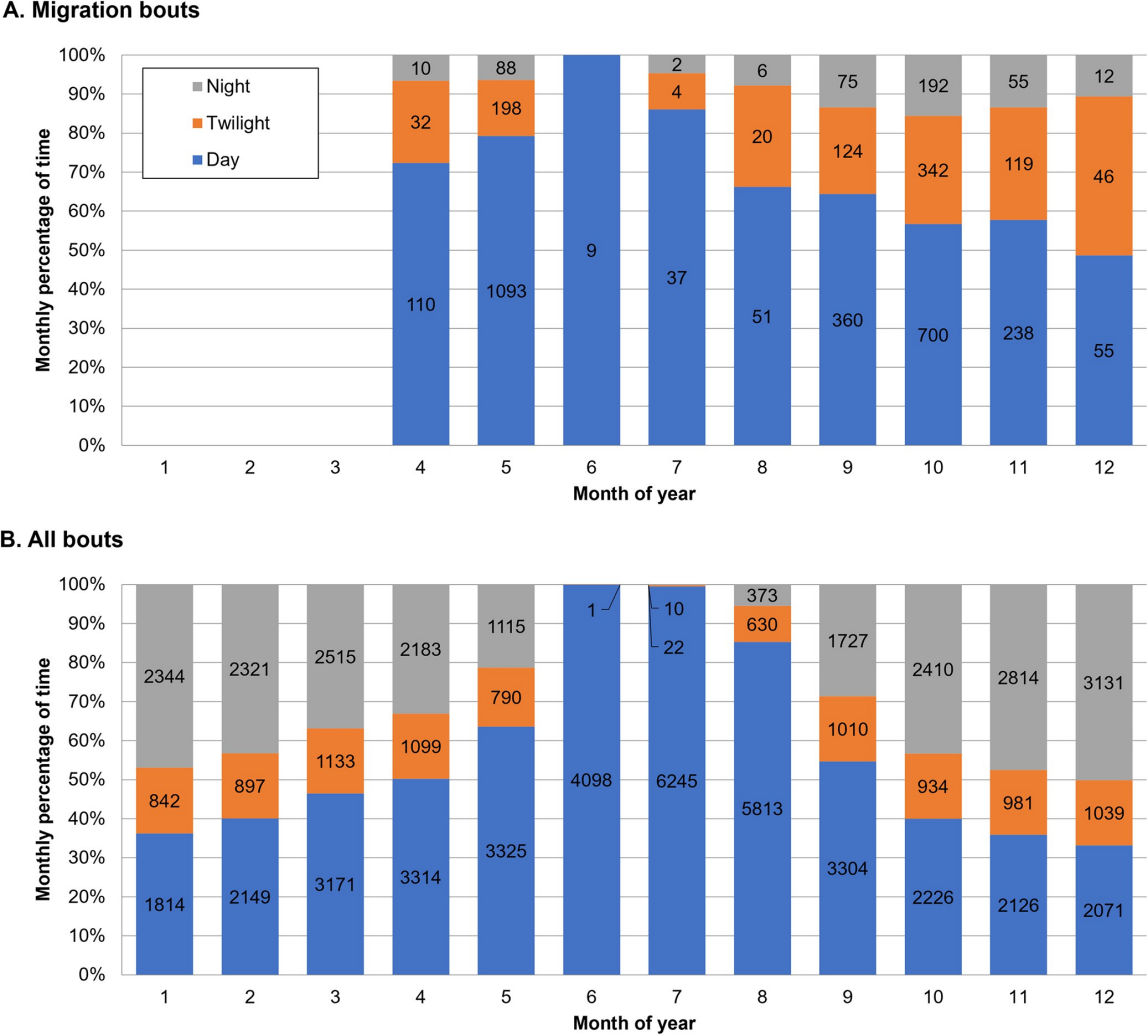
Diel patterns of 15 Vega gulls monitored in 2h bouts. Proportion of bouts is given for daylight (blue), twilight (ochre) and night (grey). Panel (A) presents the pattern for migration bouts only and must be compared with panel (B; all bouts) to infer which periods of the day are preferred for migration. The total number of recorded bouts is given in the bars.

Compared to all documented bouts, migration bouts occurred mainly during day and twilight, with only 6–7% of the bouts during the night in April-May, and 10–16% between September and December (Fig 7). Hence, although Vega gulls can migrate during the night (see also S5 Table), this does not seem to be a common behaviour.

**Fig 8:**
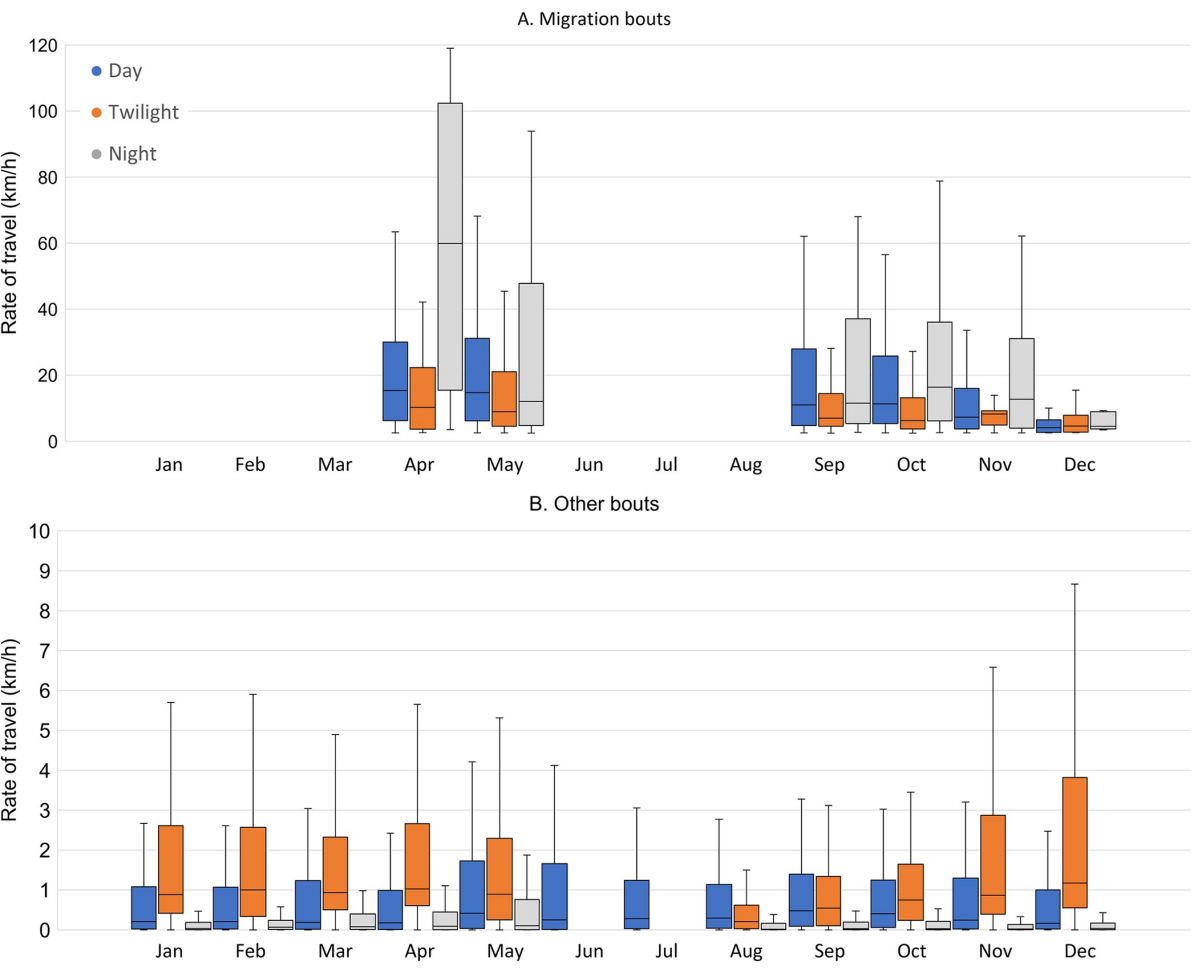
Median rates of travel (in km/h with 95% CI) of 15 Vega gulls during the day (blue), twilight (ochre) and night (grey): (A) migration bouts, (B) other bouts. Boxes hold 50% of the values, with the median shown as a horizontal line. Whiskers extend to the highest and lowest values excluding outliers.

Rates of travel were higher during spring migration (April-May) than during autumn migration (September-December), both for migration bouts (Z = 8.9; *P*<0.001; Fig 8A) and for other bouts (Z = 16; *P*<0.001; Fig 8B). During migration bouts, night bouts, although rare (Fig 7A), had higher rates of travel (and twilight bouts lower; Z = 8.4; *P*<0.001) than day bouts (Z = 2.8; *P* = 0.005). Due to smaller sample size this difference was not significant in spring (Z = 1.05; *P* = 0.29), but it was in autumn (Z = 4.4; *P*<0.001; Fig 8A). It is also worth mentioning that among the seven highest rates of speed (for 2 h bouts) recorded in this study (all between 109 and 126 km/h), five occurred during the night, one during twilight, and only one during the day. Furthermore, the 20 highest recorded rates all occurred in spring, between April 23 and May 18 (produced by eight different individuals).

Conversely, over the 12 months of the year, other bouts (Fig 8B) showed the highest rates of travel (compared to day) during twilight (Z = 44; *P*<0.001), and lowest during the night (Z = 67; *P*<0.001).

**Fig 9:**
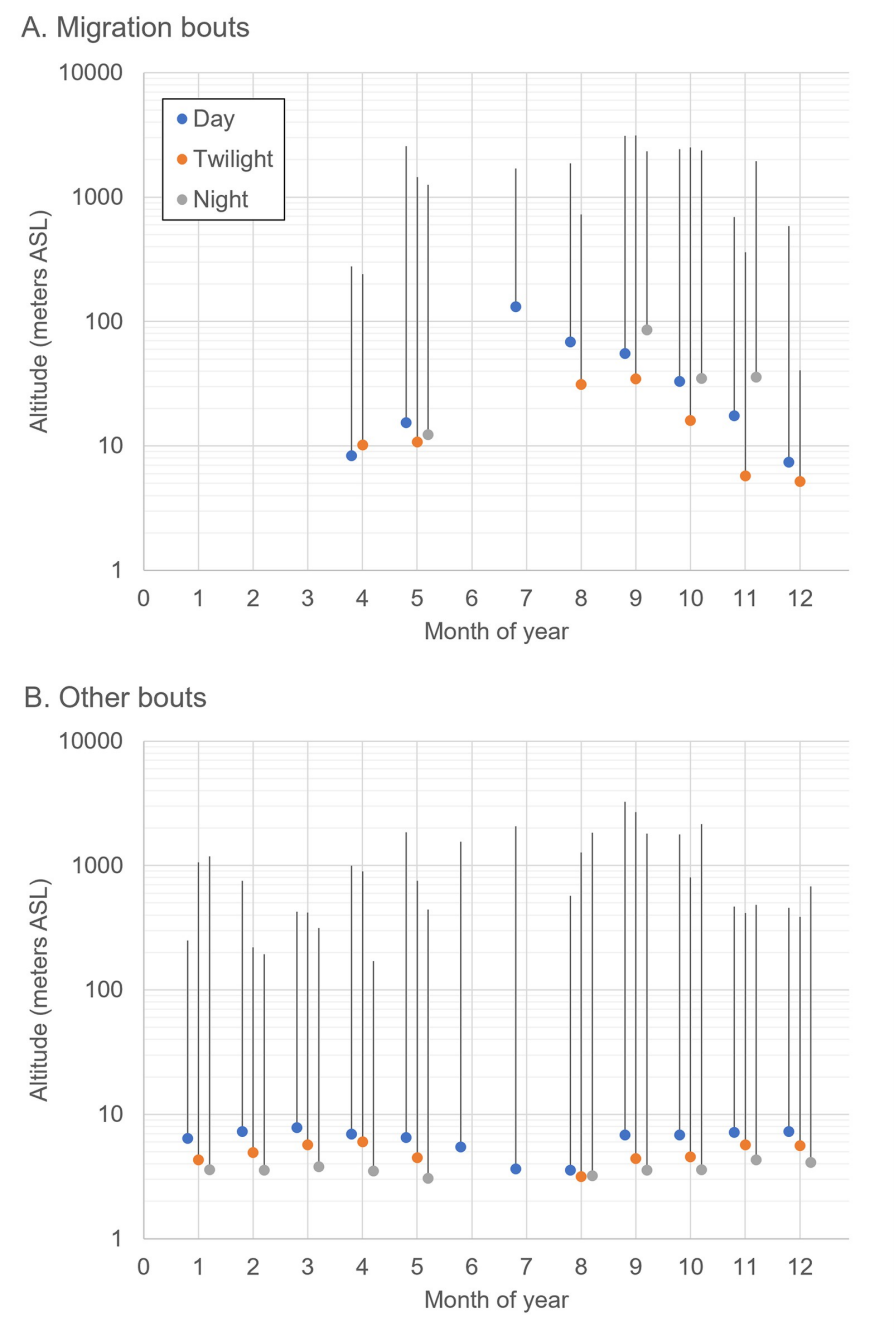
Median (dots) and maximum (lines) altitudes (in meters above sea level) of 15 Vega gulls during the day (blue), twilight (ochre) and night (grey): (A) Migration bouts, (B) Other bouts. Only medians estimated with 20 or more bouts are shown.

Overall, altitudes were higher during migration bouts (Fig 9A) than during other bouts (Fig 9B; Z = -44; *P*<0.001). This was also true when comparing monthly medians, except in November during twilight (Z = 1.47; *P* = 0.14) and in December during the day (Z = -0.61; *P* = 0.54) and twilight (Z = 0.10; *P* = 0.91). For the autumn migration bouts (September to December), median altitudes were higher for day and night flights than for twilight flights (Z = 8.25 and 4.51 respectively; both *P*<0.001), but the difference between day and night flights was not significant (Z = 0.29; *P* = 0.77; Fig 9A). During other bouts (Fig 9B), a different pattern was observed (year-round), with median altitudes higher during the day than during twilight (Z = 9.64; *P*<0.001), and lower during the night than during twilight or day (Z = 22.88 and 43.39; both *P*<0.001), although this trend was missing in August.

### Individual repeatability

**Fig 10:**
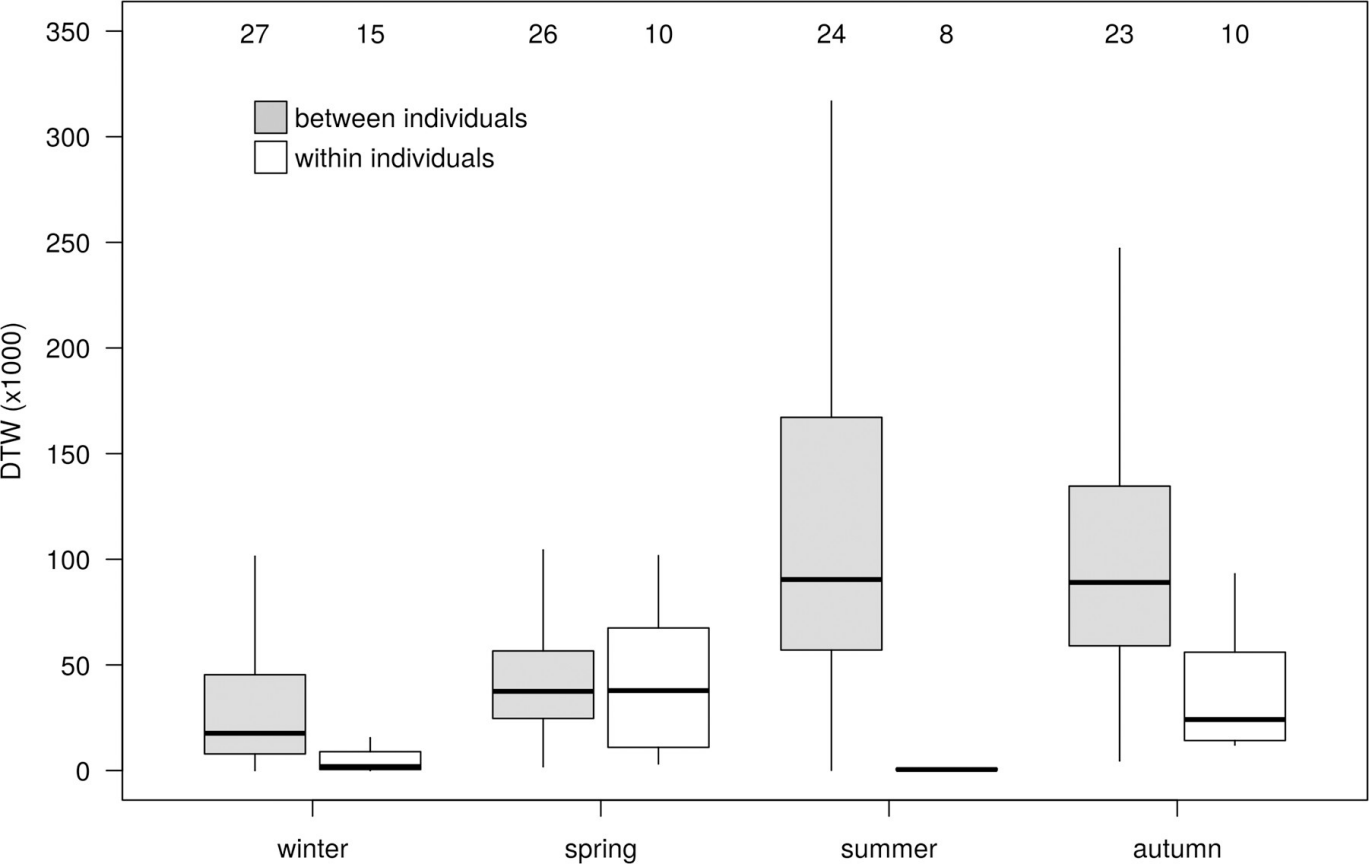
Within- and between-individual variation in movements in four seasons, as measured by Dynamic Time Warping (DTW). Boxes hold 50% of the values, with the median shown as a horizontal line. Whiskers extend to the outer 95% of the values. Sample sizes are given above the boxplots.

Dynamic Time Warping (DTW) values were significantly smaller between tracks of the same individual compared to tracks of different individuals in winter, summer and autumn (*P*<0.001), but not in spring (*P* = 0.467; Fig 10). Within individuals, DTW values in winter and summer were similarly small (*P* = 0.281), indicating very high individual consistency in both seasons. Within-individual DTW values were similarly higher during spring and autumn migration (*P* = 0.37), and significantly higher during these seasons than during winter and summer (*P*<0.001). These results indicate higher individual flexibility in migration routes and staging areas compared to the more stationary summer and winter periods. However, the individual consistency in migration routes (white bars in Fig 10) was similar between the two migration seasons, whereas inter-individual consistency (grey bars) differed between spring and autumn. Between individuals, DTW values were largest in summer, reflecting the spread of individuals over the vast breeding range.

Between subsequent years, individuals started spring migration on average within 10 days of the previous years’ date (range 1–33 d) and finished spring migration on average within three days of the previous years’ date (range 0–11 d). For the start and the end of the autumn migration, these were 13 d (range 3–32 d) and 13 d (0–26 d), respectively.

## Discussion

Hitherto, the migratory behaviour of the Vega gull had never been documented. In our study we analysed tracks of 28 Vega gulls that were all monitored for at least 100 days (mean: 383 days; Table 1). Durations of individual tracking presented in our study are longer than in most previous studies that have used the very same GPS loggers [43–46].

Overall, these birds used similar routes in spring and autumn, preferring coastal rather than inland or offshore routes (Fig 1) while travelling 4000 to 5500 km between their breeding and wintering grounds. We found no support for migratory connectivity and wintering grounds in Korea hold a mixture of birds originating from almost the entire breeding range (S3 and S4 Figs) while birds breeding in the Chaun Delta wintered both in Korea and Japan (S5 Fig). Spring migration was mostly performed in May. It was more synchronized among individuals and twice as short in terms of migration window than autumn migration (Fig 5A), the latter extending from late August to early December (Figs 2 and 3, S2 Table). However, neither the number of days spent on active migration (Fig 4) nor the mean daily distances travelled during days of active migration (Fig 6A) differed between seasons. Hence, the seasonal differences in the length of migration windows, both at individual and population levels, are mainly explained by the proportion of days “off”, with birds spending a much lower proportion (ca. twice lower) of their migration windows on active migration in autumn compared to spring migration (Fig 5B and 5C).

Both in spring and autumn, migration bouts mainly occurred during the day and twilight (Fig 7). Nevertheless, rates of travel were always higher (sometimes more than 100 km/h) during the few night flights (Fig 8A), as has also been found in Lesser Black-backed gull (*Larus fuscus*; [47]), suggesting night flights are mainly used for migratory movements. Conversely, during non-migratory bouts, rates of travel were nearly always higher during twilight than during the day or night (Fig 8B), possibly resulting from commuting flights between feeding and roosting areas, as found in Lesser Black-backed gull [48]. Flight altitudes were nearly always higher during active migration (Fig 9A), when birds also flew higher during day and night than during twilight (in line with lower rates of travel during twilight; Fig 8A), and were usually below 500 m above sea level (Fig 9A), as also reported for Lesser Black-backed gulls [47,49]. Altitudes of more than 1000 m above sea level were rare (0.28% of all positions, with the three highest between 3000–4000 m) and most (85%) were recorded in May, September and October. In several instances, these high altitudes were associated with non-stop inland flights above mountain ranges and the vast Siberian boreal forest (see e.g. S3G Fig and S5 Table), which can be seen as geographical barriers for a species normally occupying open, non-forested habitats in lowlands (see [50]). During other bouts, altitudes were always highest during the day, and lowest during the night (when most birds were probably roosting and rates of travel were also lowest; Fig 8B), except in August (Fig 9B).

When compared between years, individuals showed high consistency in their movements in winter and summer, indicating that they were site faithful to both their breeding and wintering grounds (as also evidenced for different *Larus* species in temperate regions; [51]). As expected, within-individual variation was higher during migration and similar in spring and autumn. Conversely, between individual variation was highest in summer, due to individuals spreading across a wide breeding range, and higher during the autumn than during spring migration (Fig 10).

Among the six Arctic *Larus* (see introduction), only three had been monitored by satellite or GPS tracking prior to our study, all in North America and with only 3–5 individuals tracked [8–10]. In the first study, spring migration of three adult Glaucous-winged gulls lasted for 31 days (median duration) between March 18 to April 23 (population migratory window: 37 days), autumn migration lasted for 75 days (median duration) between August 13 and December 1 (population migratory window: 111 days), and wintering grounds (located ca. 2000 km from release site) were used until mid-March [10]. In the second study, the autumn migration of four adult Thayer’s gulls was more synchronized and rapid, starting between 9–12 September and lasting for only 12–31 days (median: 21 days; population migratory window: 33 days), with birds travelling a mean distance of 3513 km [8]. In the third study, spring migration of five adult Glaucous gulls (i.e., only the GPS data of this study considered for our comparison) lasted for 15 days (median duration) between April 12 to May 24 (population migratory window: 42 days), autumn migration lasted for 46 days (median duration) between October 6 and December 27 (population migratory window: 82 days), and wintering grounds (all but one located ca. 2500 km from breeding site) were used until mid-May [9]. Compared to these three previous studies, Vega gulls showed durations of population migratory windows (mean 2015–2019: 43 days in spring and 95 days in autumn; S2 Table) similar to Glaucous-winged and Glaucous gulls. However, when considering individual migratory windows (Fig 5A), that are less sensitive to differences in methods and sample sizes, median durations (20 days in spring and 42 days in autumn; S3 Table) were lower than in Glaucous-winged gulls (31 days in spring and 75 days in autumn) despite much longer routes, similar to Glaucous gulls (15 days in spring and 46 days in autumn) and higher than in Thayer’s gull (21 days in Autumn). We suggest that the duration of migration windows in Arctic gulls could be related to the proportion of inland versus coastal habitats along their flyways, the latter providing more opportunities to forage *en route*. The flyway of the Glaucous-winged gull is exclusively coastal, allowing gulls to stop and feed almost anytime. Conversely, most of the Thayer’s gull flyway is inland, over boreal forests and mountain ranges, where habitats are less favourable for foraging [8]. Vega gulls face an intermediate situation, with ca. 25–50% of their flyways being inland in the North, depending on the exact breeding locations (Fig 1). Based on research done on North-American Herring gulls (*Larus argentatus*), Anderson et al [52] also suggested that the migration strategies of the flexible generalist *Larus* gulls may be more influenced by habitat and food resources than by migration distance.

The later spring migration in Vega and Glaucous gulls (from ca. mid-April to late May), as compared to Glaucous-winged gulls (March 18 to April 23), is most likely due to the later onset of spring in their breeding ranges where, by the end of April, the tundra is usually still covered by snow and the lakes and coast are still frozen.

Faster migration in spring than in autumn has often been reported in birds, including gulls ([9,53–56]; but see [57]), and it was suggested that this generally results from seasonal variation in stopover duration rather than from time constraints [47,58,59]. Although we did not explicitly describe stopovers in our study, the fact that Vega gulls spent a similar number of days (and had similar rates of travel) during spring and autumn migrations, while their migratory windows significantly differed in length, supports this claim and is in line with previous *Larus* studies (e.g. [47]). Although the Vega gulls must reach their breeding grounds as early as possible after snow melt to make the best use of the short Arctic summer for reproduction, their generalist and opportunistic feeding habits allow them to opt for a less time-constrained autumn migration (Fig 5), using a “fly-and-forage” migration strategy [60]. This has already been evidenced e.g. for the Glaucous gull [9] and the Lesser Black-backed gull [47], except for populations constrained by long inland flyways and significant geographical barriers, which they then tend to cross over a shorter period [57].

Launching similar studies on other Arctic gulls breeding in the more isolated (re. distance between breeding and potential wintering grounds) Western Siberia (Heuglin’s gull) or having geographically distinct populations (e.g., the Iceland gull) would help us to understand what drives the different migration strategies observed in this peculiar group of species. Follow-up analyses relating the migration behaviour of the Vega gull to breeding ecology (e.g., breeding success and phenology) and habitat selection at the wintering grounds should allow us to foresee the threats that may arise as a consequence of environmental changes and ongoing anthropic developments along the East Asian flyway.

## Supporting information

S1 Fig Seasonal changes in mean daily rates of travel. Colours indicate different duty cycles (2, 4 or 12h between fixes; ±5% to account for small variations in the duration between fixes). Since mean speeds are below 2.5 km/h for all but a few days in winter (January-March) and during early breeding season (June), we used the threshold of 60 km/h (as in as in Soriano-Redondo et al. 2020) to document the timing of migrations.
(PDF)

S2 Fig Daily latitudinal distribution (top) and daily distances travelled (bottom) by 21 Vega gulls (same individuals than on Fig 2A) between February 2015 and December 2019. The pink bars present days with at least one bird on active migration (see Methods), used to define spring and autumn migratory windows at the population level.
(PDF)

S3 Fig Individual maps for the tracks of nine adult migrating birds tagged in winter at the Samcheok site. (PDF)

S4 Fig Individual maps for the tracks of five adult migrating birds tagged in winter at the Yeongdeok site. (PDF)

S5 Fig Individual maps for the tracks of seven adult migrating birds tagged in summer at the Chaun Delta. (PDF)

S6 Fig Individual maps for the tracks of four immature and three non-breeding adults (all tagged in winter at the two Korean sites) that did not reach the species breeding range during the summer season (same individuals than on Fig 2B). (PDF)

S7 Fig Classification tree showing how the four assessment criteria (see S4 Table) were used to infer the fate of the 50 tagged Vega gulls when GPS tags stopped or were censored. (PDF)

S1 Table Individual (n = 28) monthly sample sizes available between February 2015 and December 2019. (PDF)

S2 Table Population migratory windows and periods spent on winter grounds and breeding range for the 21 Vega gulls monitored between 2015 and 2019 (same individuals than on Fig 2A). (PDF)

S3 Table Individual migratory windows for the 21 Vega gulls monitored between 2015 and 2019 (same individuals than on Fig 2A). (PDF)

S4 Table Summary table presenting details for the 50 Vega gulls tagged with GPS transmitters, the four assessment criteria used to infer their fates (see also classification presented in S7 Fig), and the three subsamples used in the different analyses. Grey lines present the 22 individuals that were monitored for less than 100 days (not included in the analyses).
(PDF)

S5 Table Examples of high altitude (i.e., >1000m) migratory flights of six Vega gulls over mountain ranges and boreal forest in North-eastern Siberia (distances and speeds estimated between current and previous positions). Note the higher speeds and relative heights reported for the night flight.
(PDF)
