# Supplementary material for: Flyways and migratory behaviour of the Vega gull (Larus vegae), a little-known Arctic endemic
Source: PLoS One. 2023 Feb 16;18(2):e0281827. doi: 10.1371/journal.pone.0281827 (PMC9934386; doi:10.1371/journal.pone.0281827)
Supplement: S1 Fig — Colours indicate different duty cycles (2, 4 or 12h between fixes; ±5% to account for small variations in the duration between fixes). Since mean speeds are below 2.5 km/h for all but a few days in winter (January-March) and during early breeding season (June), we used the threshold of 60 km/h (as in as in Soriano-Redondo et al. 2020) to document the timing of migrations. (PDF) [file pone.0281827.s001.pdf]

SUPPORTING INFORMATION

Flyways and migratory behaviour of the Vega gull (*Larus vegae*), a little-known arctic endemic

Olivier Gilg[1,2], Rob S.A. van Bemmelen[3], Hansoo Lee[4], Jin-Young Park[5], Hwa-Jung Kim[5], Dong-Won Kim[5], Won Y. Lee[6], Kristaps Sokolovskis[7] and Diana V. Solovyeva[8].

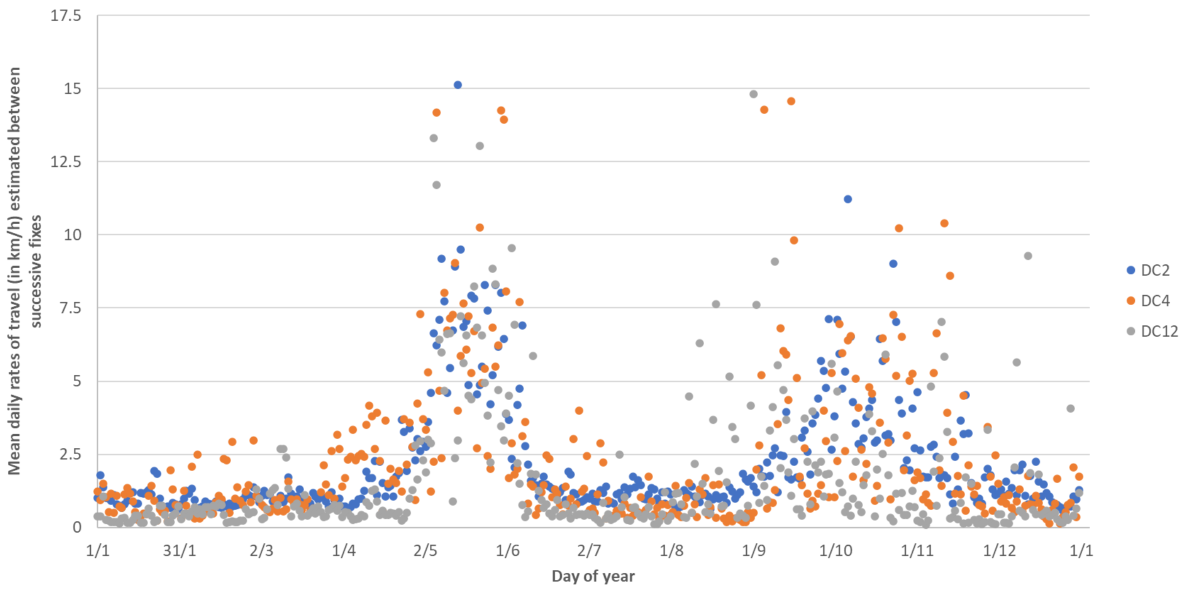

S1 Fig. Seasonal changes in mean daily rates of travel. Colours indicate different duty cycles (2, 4 or 12h between fixes; ±5% to account for small variations in the duration between fixes). Since mean speeds are below 2.5 km/h for all but a few days in winter (January-March) and during early breeding season (June), we used the threshold of 60km/h (as in Soriano-Redondo *et al.* 2020) to document the timing of migrations.
