# Supplementary material for: Flyways and migratory behaviour of the Vega gull (Larus vegae), a little-known Arctic endemic
Source: PLoS One. 2023 Feb 16;18(2):e0281827. doi: 10.1371/journal.pone.0281827 (PMC9934386; doi:10.1371/journal.pone.0281827)
Supplement: S2 Fig — Daily latitudinal distribution (top) and daily distances travelled (bottom) by 21 Vega gulls (same individuals than on Fig 2A) between February 2015 and December 2019. The pink bars present days with at least one bird on active migration (see Methods), used to define spring and autumn migratory windows at the population level. (PDF) [file pone.0281827.s002.pdf]

SUPPORTING INFORMATION

Flyways and migratory behaviour of the Vega gull (*Larus vegae*), a little-known arctic endemic

Olivier Gilg[1,2], Rob S.A. van Bemmelen[3], Hansoo Lee[4], Jin-Young Park[5], Hwa-Jung Kim[5], Dong-Won Kim[5], Won Y. Lee[6], Kristaps Sokolovskis[7] and Diana V. Solovyeva[8].

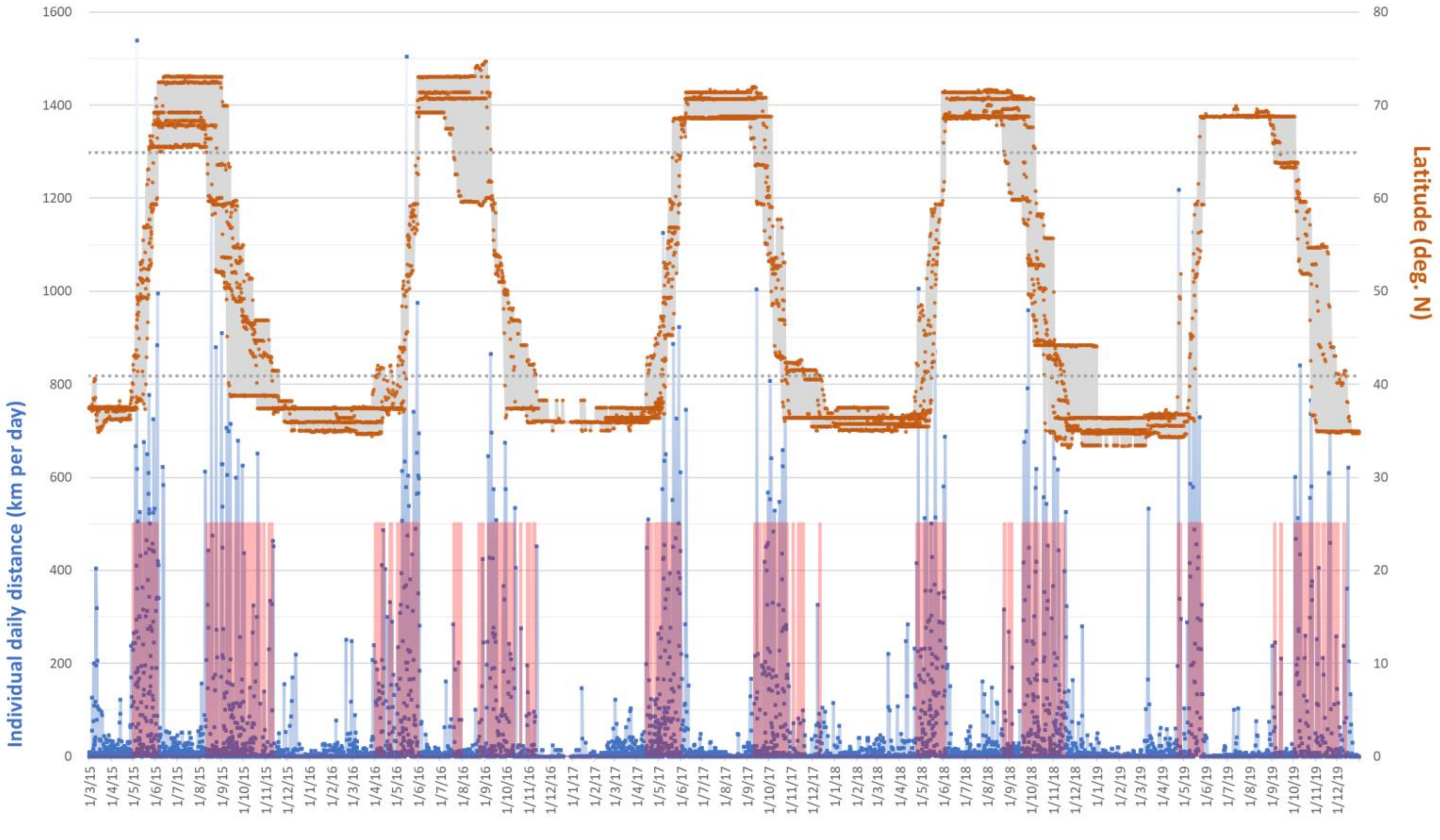

S2 Fig. Daily latitudinal distribution (top) and daily distances travelled (bottom) by 21 Vega gulls (same individuals than on Fig 2A) between February 2015 and December 2019. The pink bars present days with at least one bird on active migration (see Methods), used to define spring and autumn migratory windows at the population level.
