# Supplementary material for: Flyways and migratory behaviour of the Vega gull (Larus vegae), a little-known Arctic endemic
Source: PLoS One. 2023 Feb 16;18(2):e0281827. doi: 10.1371/journal.pone.0281827 (PMC9934386; doi:10.1371/journal.pone.0281827)
Supplement: S1 Table — (PDF) [file pone.0281827.s008.pdf]

SUPPORTING INFORMATION

Flyways and migratory behaviour of the Vega gull (*Larus vegae*), a little-known arctic endemic

Olivier Gilg[1,2], Rob S.A. van Bemmelen[3], Hansoo Lee[4], Jin-Young Park[5], Hwa-Jung Kim[5], Dong-Won Kim[5], Won Y. Lee[6], Kristaps Sokolovskis[7] and Diana V. Solovyeva[8].

	Number of different individuals monitored						No. of monthly datasets
	2015	2016	2017	2018	2019	2015-2019	2015-2019
January		5	4	6	6	15	21
February	8	7	12	6	6	26	39
March	8	7	12	6	5	26	38
April	8	7	12	6	5	26	38
May	8	7	12	6	4	26	37
June	8	5	7	7	4	25	31
July	8	5	7	7	5	24	32
August	7	5	7	7	5	23	31
September	7	5	7	7	5	23	31
October	7	5	6	7	5	22	30
November	7	5	6	6	5	20	29
December	5	4	6	6	4	17	25
Jan-Dec.	11	7	14	9	8	28	382

S1 Table. Individual (n=28) monthly sample sizes available between February 2015 and December 2019.
