# Supplementary material for: Flyways and migratory behaviour of the Vega gull (Larus vegae), a little-known Arctic endemic
Source: PLoS One. 2023 Feb 16;18(2):e0281827. doi: 10.1371/journal.pone.0281827 (PMC9934386; doi:10.1371/journal.pone.0281827)
Supplement: S2 Table — (PDF) [file pone.0281827.s009.pdf]

SUPPORTING INFORMATION

Flyways and migratory behaviour of the Vega gull (*Larus vegae*), a little-known arctic endemic

Olivier Gilg[1,2], Rob S.A. van Bemmelen[3], Hansoo Lee[4], Jin-Young Park[5], Hwa-Jung Kim[5], Dong-Won Kim[5], Won Y. Lee[6], Kristaps Sokolovskis[7] and Diana V. Solovyeva[8].

	Spring migration			Autumn migration		
Year	Start (earliest bird)	End (latest bird)	Duration (days)	Start (earliest bird)	End (latest bird)	Duration (days)
2015	1/5	3/6	34	12/8	10/11	91
2016	2/4	31/5	60	20/7	10/11	114
2017	14/4	31/5	48	12/9	11/12	91
2018	25/4	2/6	39	25/8	15/11	83
2019	24/4	26/5	33	5/9	11/12	98
Median dates & mean durations	24/4	31/5	43	25/8	15/11	95
	**Winter range (<40.9°N)**			**Summer range (>64.9°N)**		
Year	Departing (latest bird)	Arriving (earliest bird)	Duration (days)	Arriving (earliest bird)	Departing (latest bird)	Duration (days)
2015	18/5	10/9	251	23/5	9/9	110
2016	14/5	29/9	229	29/5	9/9	104
2017	10/5	19/10	204	21/5	4/10	137
2018	16/5	19/10	210	31/5	6/10	129
2019	17/5	26/10	204	23/5	2/10	133
Median dates & mean durations	16/5	19/10	220	23/5	2/10	123

S2 Table. Population migratory windows and periods spent on winter grounds and breeding range for the 21 Vega gulls monitored between 2015 and 2019 (same individuals than on Fig 2A).
