# Supplementary material for: Flyways and migratory behaviour of the Vega gull (Larus vegae), a little-known Arctic endemic
Source: PLoS One. 2023 Feb 16;18(2):e0281827. doi: 10.1371/journal.pone.0281827 (PMC9934386; doi:10.1371/journal.pone.0281827)
Supplement: S3 Table — (PDF) [file pone.0281827.s010.pdf]

SUPPORTING INFORMATION

Flyways and migratory behaviour of the Vega gull (*Larus vegae*), a little-known arctic endemic

Olivier Gilg[1,2], Rob S.A. van Bemmelen[3], Hansoo Lee[4], Jin-Young Park[5], Hwa-Jung Kim[5], Dong-Won Kim[5], Won Y. Lee[6], Kristaps Sokolovskis[7] and Diana V. Solovyeva[8].

Bird ID	Year	Spring			Autumn		
		Start (1st day)	End (last day)	Duration (days)	Start (1st day)	End (last day)	Duration (days)
bpn1721	2017				6 October	12 November	38
	2018	16 May	2 June	18			
	2019	10 May	22 May	13	3 October	25 October	23
bpn1723	2017				12 September	11 December	91
	2018	6 May	n.d.[b]				
bpn1910	2019				13 September	13 November	62
bpn1911	2019				2 October	9 November	39
br1425	2015	6 May	28 May	23	12 August	19 October	69
	2016	7 May	28 May	22	20 July	12 October	85
br1428	2015	2 May	29 May	28	10 September	n.d.[b]	
br1429	2015	7 May	22 May	16	18 August	9 September	23
	2016	9 May	n.d.[b]				
br1430	2015	1 May	22 May	22	12 August	30 October	80
br1541[a]	2016	2 April	31 May	60	6 September	10 November	66
	2017	6 May	30 May	25			
ke1512	2015	4 May	3 June	31	1 September	10 November	71
ke1514	2015	14 May	2 June	20	26 August	24 October	60
rcees1809	2018				7 October	15 November	40
	2019	9 May	22 May	14	5 September	11 December	98
rcees1811	2018				27 September	3 November	38
	2019	17 May	26 May	10			
rcees1812	2018				25 August	5 October	42
vt15079	2016	15 May	29 May	15	10 September	28 September	19
vt15081	2016	12 May	31 May	20			
vt16247	2017	7 May	20 May	14	27 September	18 October	22
	2018	6 May	21 May	16	24 September	10 October	17
vt16249	2017	9 May	30 May	22			
vt16250	2017	4 May	31 May	28	29 September	26 October	28
	2018	16 May	31 May	16	8 October	26 October	19
vt16251	2017	8 May	27 May	20			
vt16252	2017	14 April	20 May	37	14 September	26 October	43
	2018	25 April	23 May	29	21 September	12 November	53
	2019	24 April	n.d.[b]				
Median		7 May	28 May	20.00	12 September	26 October	42.00
Mean		5 May	27 May	22.57	11 September	29 October	48.96

[a] this bird left the wintering area (<40.9°Lat. N) on 2 April but stayed >43°Lat until 3 May.

[b] logger stopped before migration completed

S3 Table. Individual migratory windows for the 21 Vega gulls monitored between 2015 and 2019 (same individuals than on Fig 2A).
