# Supplementary material for: Flyways and migratory behaviour of the Vega gull (Larus vegae), a little-known Arctic endemic
Source: PLoS One. 2023 Feb 16;18(2):e0281827. doi: 10.1371/journal.pone.0281827 (PMC9934386; doi:10.1371/journal.pone.0281827)
Supplement: S4 Table — Grey lines present the 22 individuals that were monitored for less than 100 days (not included in the analyses). (PDF) [file pone.0281827.s011.pdf]

SUPPORTING INFORMATION

Flyways and migratory behaviour of the Vega gull (*Larus vegae*), a little-known arctic endemic

Olivier Gilg[1,2], Rob S.A. van Bemmelen[3], Hansoo Lee[4], Jin-Young Park[5], Hwa-Jung Kim[5], Dong-Won Kim[5], Won Y. Lee[6], Kristaps Sokolovskis[7] and Diana V. Solovyeva[8].

Trapping location and individual ID	Assessement criteria used to infer fate at end of monitoring													Subsamples[d]		
	A. Stationary bird/tag: less than 1km moved between 5 previous fixes (y/n)	B1. Expected locations recorded (%) during previous days (d)	B2. Final power supply (V)	C. Still active after 31/12/2019 (y/n)	D. Still moving (average distance between last 10 fixes; km)	Inferred fate	Age (sex from DNA when stated)	GPS logger[a]	Duty Cycle (fixes/day)	GPS Start Date (UTC+9)	GPS End Date (UTC+9)	Nb days	A (n=21)	B (n=15)	C (n=13)
Samcheok (Gangwon Province), South Korea															
ke1511	n	100%/10d	3.89	n	26.1	Logger (B2)	2nd year (male)	WT-200	2	25/02/2015	26/10/2015	243			
ke1512	n	100%/10d	3.93	n	1.7	Unknown (D)	Adult (female)	WT-200	2	25/02/2015	19/11/2015	267	X		X
ke1513	n	100%/10d	4.01	n	1.2	Unknown (D)	Adult (male)	WT-200	2	25/02/2015	22/07/2015	147			
ke1514	n	95%/10d	3.79	n	30.6	Logger (B2)	Adult (male)	WT-200	2	25/02/2015	29/10/2015	246	X		X
br1423	n	100%/10d	4.10	n	69.4	Unknown (D)[b]	Adult (male)	WT-200	2	25/02/2015	01/06/2015	96			
br1425	n	100%/10d	3.64	n	0.4	Logger (B2)	Adult (male)	WT-200 / 300	2&12	25/02/2015	15/11/2016	629	X	X	X
br1426	n	100%/10d	4.13	n	10.0	Unknown (D)	Subadult (male)	WT-200	2	25/02/2015	11/05/2015	75			
br1427	n	100%/10d	4.07	n	133.3	Unknown (D)[b]	Adult (male)	WT-200	2	25/02/2015	13/05/2015	77			
br1428	n	100%/10d	3.95	n	15.2	Unknown (D)	Adult (male)	WT-200	2	25/02/2015	02/10/2015	219	X		X
br1429	n	100%/10d	3.82	n	159.2	Logger (B2)	Adult (female)	WT-200	2	25/02/2015	11/05/2016	441	X		X
br1430	n	100%/10d	3.92	n	5.0	Unknown (D)	Adult (female)	WT-200	2	25/02/2015	22/11/2015	270	X		X
br1431	n	100%/10d	4.07	n	62.5	Unknown (D)[b]	Adult (male)	WT-200	2	25/02/2015	25/05/2015	89			
vt15079	n	100%/10d	3.91	n	4.4	Unknown (D)	Adult	WT-300	12	09/11/2015	05/05/2017	543	X	X	X
vt15080	n	58%/10d	3.54	n	0.6	Logger (B1/B2)	Adult	WT-300	12	09/11/2015	08/01/2016	60			
vt15081	n	90%/10d	4.06	n	43.1	Unknown (D)[b]	Adult	WT-300	12	09/11/2015	11/05/2017	549	X	X	
vt15088	n	100%/10d	4.12	n	19.7	Unknown (D)[b]	Adult	WT-300	12	09/11/2015	15/04/2016	158			
br1525	n	98%/10d	4.11	n	50.0	Unknown (D)[b]	1st year	WT-300 Mallard	6	16/02/2016	01/05/2016	75			
br1530	n	100%/10d	4.22	n	101.0	Unknown (D)[b]	2nd year	WT-300 Mallard	6	16/02/2016	30/04/2017	439			
br1541	n	83%/10d	3.59	n	78.1	Logger (B2)	Adult	WT-300 Mallard	6	18/02/2016	14/05/2018	816	X		X
Yeongdeok (Gyeongsangbuk Province), South Korea															
vt16247	n	92%/10d	4.13	n	22.8	Unknown (D)	Adult	WT-300	12	13/02/2017	10/10/2018	604	X	X	X
vt16248	n	100%/10d	4.23	n	0.6	Unknown (D)	Adult	WT-300	12	13/02/2017	29/04/2017	75			
vt16249	n	98%/10d	4.20	n	27.5	Unknown (D)[b]	Adult	WT-300	12	13/02/2017	30/05/2017	106	X	X	
vt16250	n	100%/10d	4.16	n	1.4	Unknown (D)	Adult	WT-300	12	13/02/2017	19/02/2019	736	X	X	X
vt16251	n	97%/10d	4.22	n	1.0	Unknown (D)[b]	Adult	WT-300	12	13/02/2017	27/05/2017	103	X	X	
vt16252	n	100%/10d	4.20	n	21.4	Unknown (D)[b]	Adult	WT-300	12	13/02/2017	27/04/2019	803	X	X	X
vt16253	n	94%/10d	4.11	n	14.2	Unknown (D)	Adult	WT-300	12	13/02/2017	14/05/2017	90			
vt16254	n	94%/10d	4.22	n	4.2	Unknown (D)[b]	Adult	WT-300	12	13/02/2017	06/06/2017	113			
vt16255	n	99%/10d	4.20	n	5.2	Unknown (D)	Adult	WT-300	12	13/02/2017	29/04/2017	75			
vt16256	n	100%/10d	4.21	n	1.9	Unknown (D)	Adult	WT-300	12	13/02/2017	16/03/2017	31			
vt16258	y	99%/10d	4.21	n	0.02	Dead or lost (A)	2nd year	WT-300	12	13/02/2017	30/03/2017	45			
vt16259	n	92%/10d	4.23	n	1.5	Unknown (D)	1st year	WT-300	12	13/02/2017	18/04/2017	64			
vt16260	n	98%/10d	4.22	n	56.1	Unknown (D)[b]	1st year	WT-300	12	13/02/2017	25/06/2017	132			
vt16261	n	100%/10d	4.01	n	0.4	Unknown (D)	1st year	WT-300	12	13/02/2017	19/09/2017	218			
Chaun Delta (Chukotka Region), Russia															
bpn1710	n	97%/10d	4.21	n	0.7	Unknown (D)	Adult	WT-300	12	23/06/2017	26/07/2017	33			
bpn1720	n	80%/10d	3.90	n	5.2	Logger (B1/B2)	Adult	WT-300	12	18/08/2017	20/10/2017	63			
bpn1721	n	99%/10d	4.15	y	2.4	Still alive (C)	Adult male	WT-300	12	24/07/2017	31/12/2019	890	X	X	X
bpn1722	n	100%/10d	4.20	n	1.7	Unknown (D)	Adult	WT-300	12	23/07/2017	02/08/2017	10			
bpn1723	n	100%/10d	4.17	n	3.3	Unknown (D)	Adult male	WT-300	12	22/07/2017	10/05/2018	292	X	X	
rcees1801	n	92%/2d	4.21	n	3.2	Unknown (D)	Adult	WT-300	12	24/06/2018	26/06/2018	2			
rcees1802	n	100%/10d	4.22	n	1.1	Unknown (D)	Adult	WT-300	12	25/06/2018	06/07/2018	11			
rcees1805	n	100%/5d	4.16	n	2.9	Unknown (D)	Adult	WT-300	12	04/09/2018	09/09/2018	5			
rcees1806	n	100%/8d	4.14	n	2.6	Unknown (D)	Adult	WT-300	12	02/09/2018	10/09/2018	8			
rcees1808	n	100%/1d	4.23	n	0.9	Unknown (D)	Adult	WT-300	12	25/06/2018	26/06/2018	1			
rcees1809	n	100%/10d	4.16	n	1.7	Still alive (C)	Adult female	WT-300	12	28/06/2018	31/12/2019	551	X	X	X
rcees1810	n	100%/10d	4.22	n	1.2	Unknown (D)	Adult	WT-300	12	25/06/2018	19/07/2018	24			
rcees1811	n	100%/10d	4.14	n	2.4	Unknown (D)[b]	Adult female	WT-300	12	28/06/2018	29/05/2019	335	X	X	
rcees1812	n	100%/10d	4.18	y	3.0	Still alive (C)	Adult male	WT-300	12	28/06/2018	31/12/2019	551	X	X	
bpn1908	n	100%/4d	4.15	n	0.09	Unknown (D)	Adult	WT-300 Buzzard	12	23/06/2019	27/06/2019	4			
bpn1910	n	73%/10d	3.90	n	3.0	Logger (B1)	Adult female	WT-300 Buzzard	12	23/06/2019	14/11/2019	144	X	X	
bpn1911	n	26%/10d	3.91	y	6.0	Still alive (C)	Adult female	WT-300 Buzzard	12	23/06/2019	31/12/2019	191	X	X	

[a] KoEco WT-200: 57g battery powered; KoEco WT-300: 42g solar; KoEco WT-300 Buzzard: 34g solar; KoEco WT-300 Mallard: 27g solar.

[b] for 13 of the birds with unknown fate, loggers stopped transmitting during Spring migration, when entering remote Siberian regions with poor GSM-GPRS coverage

[c] additional locations collected after this date but not included in this study

[d] list of birds used in different analyses: A= 21 birds with migration data from wintering to summering sites, B= 15 of the birds from subsample A with a 2h duty cycle, C= 13 of of the birds from subsample A which have produced data for both Spring and Autumn migration (see Methods)

S4 Table. Summary table presenting details for the 50 Vega gulls tagged with GPS transmitters, the four assessment criteria used to infer their fates (see also classification presented in Figure S7)**, and the three subsamples used in the different analyses**. Grey lines present the 22 individuals that were monitored for less than 100 days (not included in the analyses).
