# Supplementary material for: Flyways and migratory behaviour of the Vega gull (Larus vegae), a little-known Arctic endemic
Source: PLoS One. 2023 Feb 16;18(2):e0281827. doi: 10.1371/journal.pone.0281827 (PMC9934386; doi:10.1371/journal.pone.0281827)
Supplement: S5 Table — Note the higher speeds and relative heights reported for the night flight. (PDF) [file pone.0281827.s012.pdf]

SUPPORTING INFORMATION

Flyways and migratory behaviour of the Vega gull (*Larus vegae*), a little-known arctic endemic

Olivier Gilg[1,2], Rob S.A. van Bemmelen[3], Hansoo Lee[4], Jin-Young Park[5], Hwa-Jung Kim[5], Dong-Won Kim[5], Won Y. Lee[6], Kristaps Sokolovskis[7] and Diana V. Solovyeva[8].

Bird ID	Date (UTC+9)	Decimal Latitude	Decimal Longitude	Distance (km)	Speed (km/h)	Day or Night	Bird Altitude (m)	Ground elevation (m)	Bird relative height (m)
bpn1721	2019-05-22 04:00:45	61.704	157.373	20.78	10.4	D	77	0	77
bpn1721	2019-05-22 06:00:45	62.029	157.835	43.54	21.8	D	791	202	589
bpn1721	2019-05-22 08:00:40	62.774	158.817	96.98	48.5	D	1510	1234	276
bpn1721	2019-05-22 10:00:45	63.495	159.418	85.65	42.8	D	1825	837	988
bpn1721	2019-05-22 12:00:43	64.182	160.379	89.71	44.9	D	1657	707	950
bpn1721	2019-05-22 14:00:42	64.731	161.567	83.41	41.7	D	1674	658	1016
bpn1721	2018-06-02 08:00:45	62.156	158.136	50.82	25.4	D	1271	911	360
bpn1721	2018-06-02 10:00:45	62.662	159.179	77.73	38.9	D	1447	550	897
bpn1721	2018-06-02 12:00:45	63.287	159.961	79.93	40.0	D	1214	880	334
bpn1721	2018-06-02 14:00:40	63.873	160.723	75.27	37.7	D	975	840	135
bpn1721	2018-06-02 16:00:45	64.535	161.365	79.81	39.9	D	805	731	74
bpn1721	2018-10-13 22:00:40	64.684	166.129	137.70	68.9	N	2270	633	1637
bpn1721	2018-10-14 00:00:45	63.571	164.820	139.09	69.5	N	2406	599	1807
bpn1721	2018-10-14 02:00:40	62.408	163.180	153.53	76.8	N	1546	68	1478
bpn1721	2018-10-14 04:00:40	61.442	161.521	137.96	69.0	N	3134	170	2964
br1425	2016-05-27 06:31:20	59.847	144.598	52.43	26.1	D	1742	517	1225
br1425	2016-05-27 08:30:57	60.427	144.978	67.87	34.0	D	930	568	362
br1425	2016-05-27 10:30:45	61.065	144.836	71.32	35.7	D	1408	635	773
br1425	2016-05-27 12:31:12	61.821	145.257	86.95	43.3	D	1809	1093	716
br1425	2016-05-27 14:30:38	62.380	146.023	73.74	37.0	D	1322	899	423
br1425	2016-05-27 16:30:48	62.871	146.628	62.73	31.3	D	1282	958	324
br1425	2016-05-27 18:31:01	63.348	147.510	69.13	34.5	D	990	893	97
br1425	2016-05-27 20:30:43	63.460	147.830	20.18	10.1	D	940	927	13
br1425	2016-05-28 02:31:33	64.148	148.397	76.69	38.2	D	1994	1052	942
br1425	2016-05-28 04:30:45	64.876	149.599	99.20	49.9	D	1420	694	726
vt15079	2016-05-28 14:30:43	58.274	140.229	30.91	15.5	D	764	401	363
vt15079	2016-05-28 16:30:42	59.041	139.366	98.73	49.4	D	997	536	461
vt15079	2016-05-28 18:30:43	59.784	138.040	111.54	55.8	D	1070	694	376
vt15079	2016-05-28 20:30:32	60.665	137.441	103.39	51.8	D	1220	889	331
vt15079	2016-05-28 22:30:26	61.680	136.403	125.69	62.9	D	1391	328	1063
vt15079	2016-05-29 00:30:42	62.679	136.258	111.27	55.5	D	708	169	539
vt15079	2016-05-29 02:30:27	63.523	136.333	93.97	47.1	D	1272	645	627
vt15079	2016-05-29 04:30:48	64.431	135.914	102.91	51.3	D	864	481	383
vt15079	2016-09-09 06:30:27	62.035	152.369	137.31	69.0	D	2434	951	1483
vt15079	2016-09-09 08:30:42	60.995	152.571	116.12	57.9	D	2368	668	1700
vt15079	2016-09-09 10:30:37	60.394	152.758	67.58	33.8	D	2007	795	1212
vt15079	2016-09-09 12:30:38	59.708	152.760	76.24	38.1	D	667	382	285
vt15079	2016-09-09 14:30:32	59.355	152.534	41.18	20.6	D	86	94	(0)
vt15081	2016-05-30 08:30:58	60.606	144.947	72.32	36.5	D	436	376	60
vt15081	2016-05-30 10:31:18	61.294	144.866	76.55	38.2	D	2084	1174	910
vt15081	2016-05-30 12:31:13	61.993	145.046	78.29	39.2	D	1815	1385	430
vt15081	2016-05-30 14:30:43	62.517	145.694	67.17	33.7	D	2402	1446	956
vt15081	2016-05-30 16:31:23	63.073	146.338	69.92	34.8	D	1328	808	520
vt15081	2016-05-30 18:31:13	63.728	146.899	77.98	39.0	D	1564	1093	471
vt15081	2016-05-30 20:31:23	64.314	146.893	65.12	32.5	D	1230	1228	2
vt16247	2018-09-24 06:00:45	61.884	152.121	47.91	24.0	D	1481	827	654
vt16247	2018-09-24 08:00:46	60.963	151.776	103.94	52.0	D	1343	856	487
vt16247	2018-09-24 10:00:46	60.356	151.760	67.51	33.8	D	1368	688	680
vt16247	2018-09-24 12:01:05	60.035	151.638	36.25	18.1	D	205	204	1
vt16250	2017-05-30 04:00:45	60.090	143.133	77.81	38.9	D	1352	440	912
vt16250	2017-05-30 06:00:45	61.218	143.072	125.47	62.7	D	2048	1480	568
vt16250	2017-05-30 08:00:45	62.272	143.810	123.37	61.7	D	2238	1525	713
vt16250	2017-05-30 10:00:54	62.894	144.458	76.66	38.3	D	2559	877	1682
vt16250	2017-05-30 12:00:45	63.543	145.520	89.55	44.8	D	1818	936	882
vt16250	2017-05-30 14:00:45	63.793	145.629	28.32	14.2	D	793	781	12
vt16250	2017-05-30 16:00:45	63.889	145.820	14.24	7.1	D	1072	861	211
vt16250	2017-05-30 18:00:54	64.474	146.942	84.71	42.3	D	1448	1418	30
vt16250	2017-05-30 20:00:45	64.560	147.149	13.77	6.9	D	997	993	4
vt16250	2018-05-30 14:00:57	60.010	142.357	47.52	23.8	D	1056	412	644
vt16250	2018-05-30 16:01:15	60.313	141.812	45.12	22.5	D	1106	434	672
vt16250	2018-05-30 18:00:55	60.627	141.362	42.72	21.4	D	1927	789	1138
vt16250	2018-05-30 20:00:56	60.828	141.269	22.92	11.5	D	750	721	29
vt16250	2018-05-31 04:00:56	61.217	140.783	50.46	25.3	D	2308	802	1506
vt16250	2018-05-31 06:00:45	61.816	141.070	68.28	34.2	D	1614	1212	402
vt16250	2018-05-31 08:01:14	62.490	140.788	76.36	38.0	D	2148	1792	356
vt16250	2018-05-31 10:00:45	62.361	141.034	19.17	9.6	D	2168	1500	668
vt16250	2018-05-31 12:01:06	62.975	141.832	79.46	39.6	D	1739	968	771
vt16250	2018-05-31 14:00:45	63.549	141.943	64.04	32.1	D	1603	728	875
vt16250	2018-05-31 16:00:55	64.128	142.254	66.10	33.0	D	1883	1078	805
vt16250	2018-05-31 18:01:04	64.606	143.157	68.65	34.3	D	1870	938	932

S5 Table. Examples of high altitude (i.e., >1000m) migratory flights of six Vega gulls over mountain ranges and boreal forest in North-eastern Siberia (distances and speeds estimated between current and previous positions). Note the higher speeds and relative heights reported for the night flight.
